# βAR-mTOR-lipin1 pathway mediates PKA-RIIβ deficiency-induced adipose browning

**DOI:** 10.7150/thno.97046

**Published:** 2024-08-26

**Authors:** Bingwei Wang, Zhiping Hu, Long Cui, Miao Zhao, Zhijie Su, Yong Jiang, Jiarui Liu, Yun Zhao, Yujia Hou, Xiaoning Yang, Chenyu Zhang, Bingbing Guo, Daotong Li, Liang Zhao, Shengmin Zheng, Yiguo Zhao, Weipeng Yang, Dunfang Wang, Siwang Yu, Shigong Zhu, Yi Yan, Geheng Yuan, Kailong Li, Wenqiang Zhang, Lihua Qin, Weiguang Zhang, Feng Sun, Jianyuan Luo, Ruimao Zheng

**Affiliations:** 1 Department of Anatomy, Histology and Embryology, School of Basic Medical Sciences, Peking University, Beijing, China.; 2 Basic Medicine Research Innovation Center for Cardiometabolic Diseases, Ministry of Education, Southwest Medical University, Luzhou, China.; 3 Department of Hepatobiliary Surgery, Peking University People's Hospital, Peking University, Beijing, China.; 4 Department of General Surgery, Peking University Third Hospital, Peking University, Beijing, China.; 5 Department of General Surgery, Peking University First Hospital, Peking University, Beijing, China.; 6 National Engineering Research Center for Fruit and Vegetable Processing, Key Laboratory of Fruits and Vegetables Processing, College of Food Science and Nutritional Engineering, Ministry of Agriculture, Engineering Research Centre for Fruits and Vegetables Processing, Ministry of Education, China Agricultural University, Beijing, China.; 7 Department of Obstetrics and Gynecology, Beijing Jishuitan Hospital, Peking University, Beijing, China.; 8 Department of Gastrointestinal Surgery, Peking University International Hospital, Peking University, Beijing, China.; 9 Institute of Chinese Materia Medica, China Academy of Chinese Medical Sciences, Beijing, China.; 10 State Key Laboratory of Natural and Biomimetic Drugs, Department of Molecular and Cellular Pharmacology, Peking University, Beijing, China.; 11 Department of Physiology and Pathophysiology, School of Basic Medical Sciences, Peking University, Beijing, China.; 12 Department of Sport Biochemistry, School of Sport Science, Beijing Sport University, Beijing, China.; 13 Department of Endocrinology, Peking University First Hospital, Beijing, China.; 14 Department of Biochemistry and Biophysics, School of Basic Medical Sciences, Peking University Health Science Center, Beijing, China.; 15 College of Engineering, China Agricultural University, Beijing, China.; 16 Department of Epidemiology and Biostatistics, School of Public Health, Peking University, Beijing, China.; 17 Department of Medical Genetics, School of Basic Medical Sciences, Peking University, Beijing, China.; 18 Department of Biochemistry and Molecular Biology, School of Basic Medical Sciences, Peking University, Beijing, China.; 19 Neuroscience Research Institute, Peking University, Beijing, China.; 20 Key Laboratory for Neuroscience, Ministry of Education/National Health Commission, Peking University, Beijing, China.; 21 Beijing Life Science Academy, Beijing, China; 22 Present address: Department of Pathology, University of Pittsburgh School of Medicine, Pittsburgh, PA 15261, USA.

**Keywords:** Protein Kinase A, Sympathetic nerves, mTOR, Lipin1, White adipose browning

## Abstract

**Background:** Enhancing white adipose tissue (WAT) browning combats obesity. The RIIβ subunit of cAMP-dependent protein kinase (PKA) is primarily expressed in the brain and adipose tissue. Deletion of the hypothalamic RIIβ gene centrally induces WAT browning, yet the peripheral mechanisms mediating this process remain unexplored.

**Methods:** This study investigates the mechanisms underlying WAT browning in RIIβ-KO mice. Genetic approaches such as β3-adrenergic receptors (β3ARs) deletion and sympathetic denervation of WAT were utilized. Genome-wide transcriptomic sequencing and bioinformatic analysis were employed to identify potential mediators of WAT browning. siRNA assays were employed to knock down mTOR and lipin1 *in vitro*, while AAV-shRNAs were used for the same purpose *in vivo*.

**Results:** We found that WAT browning substantially contributes to the lean and obesity-resistant phenotypes of RIIβ-KO mice. The WAT browning can be dampened by β_3_ARs deletion or WAT sympathetic denervation. We identified that adipocytic mTOR and lipin1 may act as mediators of the WAT browning. Inhibition of mTOR or lipin1 abrogates WAT browning and hinders the lean phenotype of RIIβ-KO mice. In human subcutaneous white adipocytes and mouse white adipocytes, β_3_AR stimulation can activate mTOR and causes lipin1 nuclear translocation; knockdown of mTOR and Lipin1 mitigates WAT browning-associated gene expression, impedes mitochondrial activity. Moreover, mTOR knockdown reduces lipin1 level and nuclear translocation, indicating that lipin1 may act downstream of mTOR. Additionally, *in vivo* knockdown of mTOR and Lipin1 diminished WAT browning and increased adiposity.

**Conclusions:** The β_3_AR-activated mTOR-lipin1 axis mediates WAT browning, offering new insights into the molecular basis of PKA-regulated WAT browning. These findings provide potential adipose target candidates for the development of drugs to treat obesity.

## Introduction

Obesity is a chronic, multifactorial disease characterized by excessive fat storage, resulting from complex interactions between environmental and genetic factors 1-3. Growing evidence indicates that obesity, as a primary risk factor, is closely associated with type 2 diabetes, hypertension, cardiovascular disease, and reduced lifespan 4-6. Obesity has become one of the most significant public health challenges in the 21^st^ century 1, 7. Therefore, there is a high unmet need for the treatment of obesity 8, 9. A new promising strategy for the prevention and treatment of obesity is to enhance the energy expenditure (EE) in the metabolic organs, such as white adipose tissues (WATs) 10-12.

The diversity of G protein-coupled receptors (GPCRs) signal transduction arises from various interaction between GPCRs and other cell signal factors, leading to distinct downstream effects 13-17. This diversity allows for fine-tuned control over lipid metabolism processes, such as lipid mobilization and lipolysis, in response to various physiological needs upon external stimuli 16, 18, 19. The role of sympathetic nervous system (SNS) in regulating lipid metabolism has garnered significant attention 7, 20. Lipid catabolism involves various signaling pathways, with a notable focus on GPCRs such as β-adrenergic receptors (βAR) 21. βAR is important in mediating the effects of neurotransmitters that regulate lipid metabolism 21-23. Nevertheless, despite these advancements, the signal factors mediating adipocytic βAR signaling and lipid catabolism remain incompletely understood.

Stimulating the development of beige adipocytes in WAT, also known as 'WAT browning', can confer the characteristics of brown adipose tissue (BAT, a crucial thermogenic tissue) on WAT, leading to the manifestation of the catabolic state, such as increased EE, enhanced thermogenesis and reduced adiposity in mice 24, 25. In humans and rodents, there are substantial WAT depots showing potential for browning, although their abundance has been reported to be diminished in older and obese subjects 25, 26. RIIβ-PKA is tightly linked to the regulation of fat homeostasis 27, 28. Knockout of hypothalamic RIIβ subunit gene centrally leads to WAT browning and leanness 27, 28. The hypothalamus is a key brain region that drives the sympathetic nerve activity to control WAT browning 29-31. However, the peripheral mechanisms mediating adipocytic βAR signaling and the WAT browning remain to be determined.

The PKA plays a major role in the regulation of metabolism 32-37. The PKA holoenzyme is a heterotetramer composed of two homodimeric regulatory (R) subunits combined with two catalytic (C) subunits 38. Four regulatory (RIα, RIβ, RIIα, RIIβ) and two catalytic (Cα, Cβ) isoform genes have been described in the mouse 38, 39. The PKA-RIIβ subunit (RIIβ subunit) is highly expressed in the brain and adipose tissue, with limited expression elsewhere 38-40. Knockout of the RIIβ subunit gene leads to lean and healthy metabolic phenotypes in mice, exhibiting a 50% reduction in whole-body adiposity, lowered body weight, robust WAT browning, elevated EE, and are resistant to diet-induced obesity (DIO) and diabetes 27, 28, 39, 41. These aforementioned phenotypes can be rescued by RIIβ subunit gene re-expression in hypothalamus of RIIβ-KO mice; and hypothalamic RIIβ-PKA regulates WAT browning 27, 28. Of note, the WAT browning essentially contributes to the lean and obesity-resistant phenotypes of RIIβ-KO mice 28, 42. Physiologically, the hypothalamus governs WAT browning via hypothalamic-SNS axis 43-45. Enhanced SNS outflow to the adipose tissues elicits WAT browning through βAR signal to reduce adiposity 44. Cold exposure increases sympathetic outflow to WAT to promote adipose browning via βAR signal, and genetic deletion of βAR diminishes this cold-induced WAT browning 12, 16, 46. A series of adipocytic factors activated by βAR signal have been identified to be involved in the mediation of WAT browning. β_3_AR signaling was reported to activates the spermidine/spermine N1-acetyltransferase (SAT1) in adipocytes to promote WAT browning and prevent high-fat diet (HFD)-induced obesity 47. Cold exposure activates the adipocytic lysine-specific demethylase 1 (LSD1) to induce WAT browning via sympathetic nerves 48. Nevertheless, the intracellular signaling pathways that mediate WAT browning remain incompletely understood.

In white adipocytes, the mammalian target of rapamycin (mTOR), a conserved serine/threonine protein kinase, acts as a key hub to coordinate both anabolic and catabolic processes 49. The overall abundance of intracellular mTOR may be subject to pathophysiological states or specific genetic interventions 50-52. mTOR-containing protein complex-1 (mTORC1) is involved in promoting insulin-induced lipid storage and adipose expansion; whereas, mTOR is also required for WAT browning induced by βAR activation 49, 53-55. Lipin1, as an enzyme that can catalyze the phosphatidic acids to form diacylglycerols, it also functions as a coregulator of DNA-bound transcription factors 56-58. Lipin1 can translocate from the cytosol to the nucleus to govern the adipocyte differentiation and fat metabolism 56, 58. Mice with adipocyte-specific expression of a truncated lipin1 retaining transcriptional regulatory function but lacking enzyme activity exhibit diminished adiposity 57. Lipin1 expression can be induced by cold exposure and contributes to thermogenesis of adipose tissue 59. In the current work, the genome-wide transcriptomic analysis and western analysis showed heightened levels of mTOR and lipin1 in WAT of RIIβ-KO mice. However, whether mTOR and lipin1 are involved in the mediation of PKA-regulated WAT browning remains unknown.

To investigate the molecular mechanism mediating the WAT browning of RIIβ-KO mice, a series of genetically modified mouse strains, genome-wide transcriptome sequencing, sympathetic denervation, human primary adipocyte isolation and culture, RNAi-mediated in vitro RNA interference, adeno-associated virus (AAV)-mediated in vivo RNA interference, and metabolic measurements were employed in this study. Our findings reveal that β_3_AR-mTOR-lipin1 axis may serve as a key adipocytic molecular pathway for mediating WAT browning, and also provide a novel insight into the mechanism underlying the regulation of adipose metabolic homeostasis.

## Results

### PKA-RIIβ subunit deficiency can enhance white fat browning at thermoneutrality

**Figure 1:**
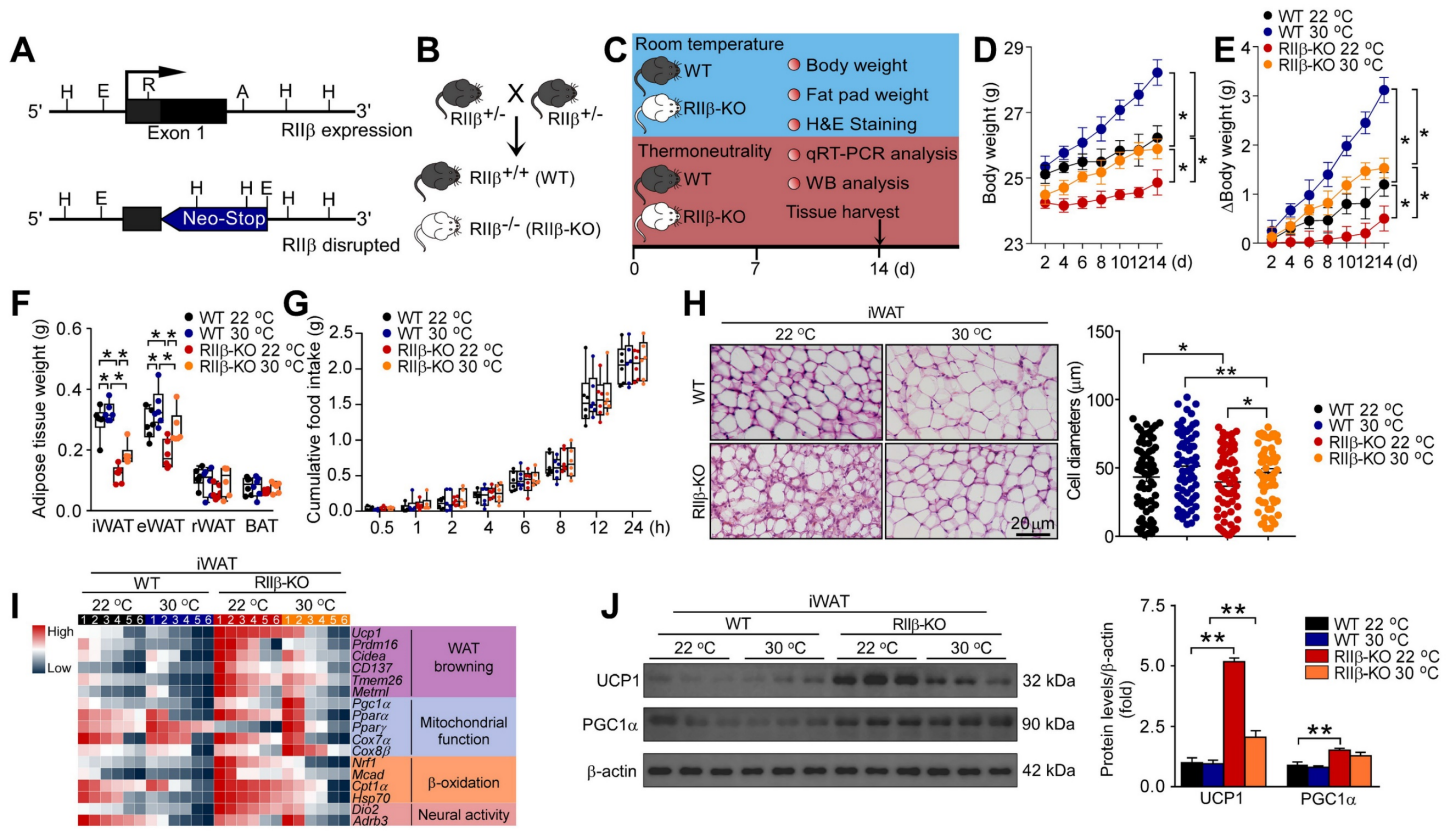
** PKA-RIIβ deficiency enhances WAT browning at room temperature or thermoneutrality. (A)** Schematic illustration of the targeting strategy for generating RIIβ-knockout mice. The targeting vector replaces the coding region of exon 1 of the RIIβ gene with a neomycin resistance cassette (Neo). Restriction enzyme sites shown include the following: A, *AatII*; E, *EcoRI*; H, *HindIII*; R, *RsrI*. **(B)** Breeding strategy for generation of RIIβ^-/-^ mice (RIIβ-KO). **(C)** Schematic illustration of experiments. Mice were kept at thermoneutrality for 14 consecutive days. Tissues were harvested for molecular analyses on day 14. **(D)** Body weight. **(E)** Increase of body weight. **(F)** Fat-pad weight. **(G)** Cumulative food intake. WT 22 °C n = 6; RIIβ-KO 22 °C n = 6; WT 30 °C n = 6; RIIβ-KO 30 °C n = 6. Values show mean ± SEM. **(H)** Representative images of H&E staining of iWAT and the size profiling of adipocytes from iWAT. Scale bar indicates 20 μm. **(I)** Heatmap shows mRNA levels of WAT browning associated genes in iWAT. The data for the heatmap was adjusted using a log2 base for normalized values. A relative color scheme uses the minimum and maximum values in each row to convert values to colors. **(J)** Representative immunoblots of UCP1, PGC1α and β-actin from iWAT, and the quantified ratio of UCP1/β-actin, PGC1α/β-actin. P values were determined by two-way ANOVA followed by Tukey's multiple comparisons test. *P < 0.05 and **P < 0.01.

To determine whether the deficiency of RIIβ induces WAT browning, we generated RIIβ^-/-^ mice (RIIβ-KO) mice. RIIβ knockout and breeding strategy to generate RIIβ-KO mice were illustrated in Figure 1A-B. To observe whether the WAT browning phenotype is present in RIIβ-KO mice, the histological and molecular properties of WAT browning were examined (Figure 1C). In order to rule out the promoting effects of environmental temperature on WAT browning 60, 61, the experiments were initially performed at both thermoneutrality (30 °C) and room temperature (22 °C) (Figure 1C). We found that the body weight (WT 22 °C, 26.2 ± 0.4 g; WT 30 °C, 28.2 ± 0.4 g; RIIβ-KO 22 °C, 24.9 ± 0.4 g, and RIIβ-KO 30 °C, 25.9 ± 0.3 g) and the fat pad weight of inguinal white adipose tissue (iWAT, WT 22 °C, 0.31 ± 0.03 g; WT 30 °C, 0.35 ± 0.01 g; RIIβ-KO 22 °C, 0.14 ± 0.01 g, and RIIβ-KO 30 °C, 0.22 ± 0.02 g) and the fat pad weight of epididymal WAT (eWAT, WT 22 °C, 0.32 ± 0.01 g; WT 30 °C, 0.34 ± 0.04 g; RIIβ-KO 22 °C, 0.22 ± 0.01 g, and RIIβ-KO 30 °C, 0.30 ± 0.03 g) of RIIβ-KO mice were lower than that of control mice (Figure 1D-F). Moreover, the body weight and adiposity of RIIβ-KO and WT mice in thermoneutrality were markedly higher than that of controls at room temperature (Figure 1D-F). The cumulative food intake did not differ between RIIβ-KO mice and WT mice (Figure 1G). The abundant multilocular lipid droplets were observed in iWAT of RIIβ-KO mice, as compared with control mice (Figure 1H). The expression levels of WAT browning-associated genes including *Ucp1, Prdm16, Cidea, CD137, Tmem26,* and *Metrnl*; and mitochondrial function-related genes, including *Pgc1α, PPARα, Cox7α1, Cox8β, Nrf1, Mcad, Cpt1α*, and *HSP7* were remarkably increased in iWAT of RIIβ-KO mice (Figure 1I). In addition, the protein levels of UCP1, the canonical marker of WAT browning; and PGC1α, the regulator of mitochondrial biogenesis and function, were elevated as compared with controls (Figure 1J). Taken together, these results uncovered the morphological feature and molecular signature of WAT browning in RIIβ-KO mice at thermoneutrality, determining the existence of fat browning in the RIIβ-KO mice; showing that brown fat-like energy expenditure phenotype may contribute to the reduced adiposity of RIIβ-KO mice.

### PKA-RIIβ subunit deficiency elevates WAT sympathetic activity

**Figure 2:**
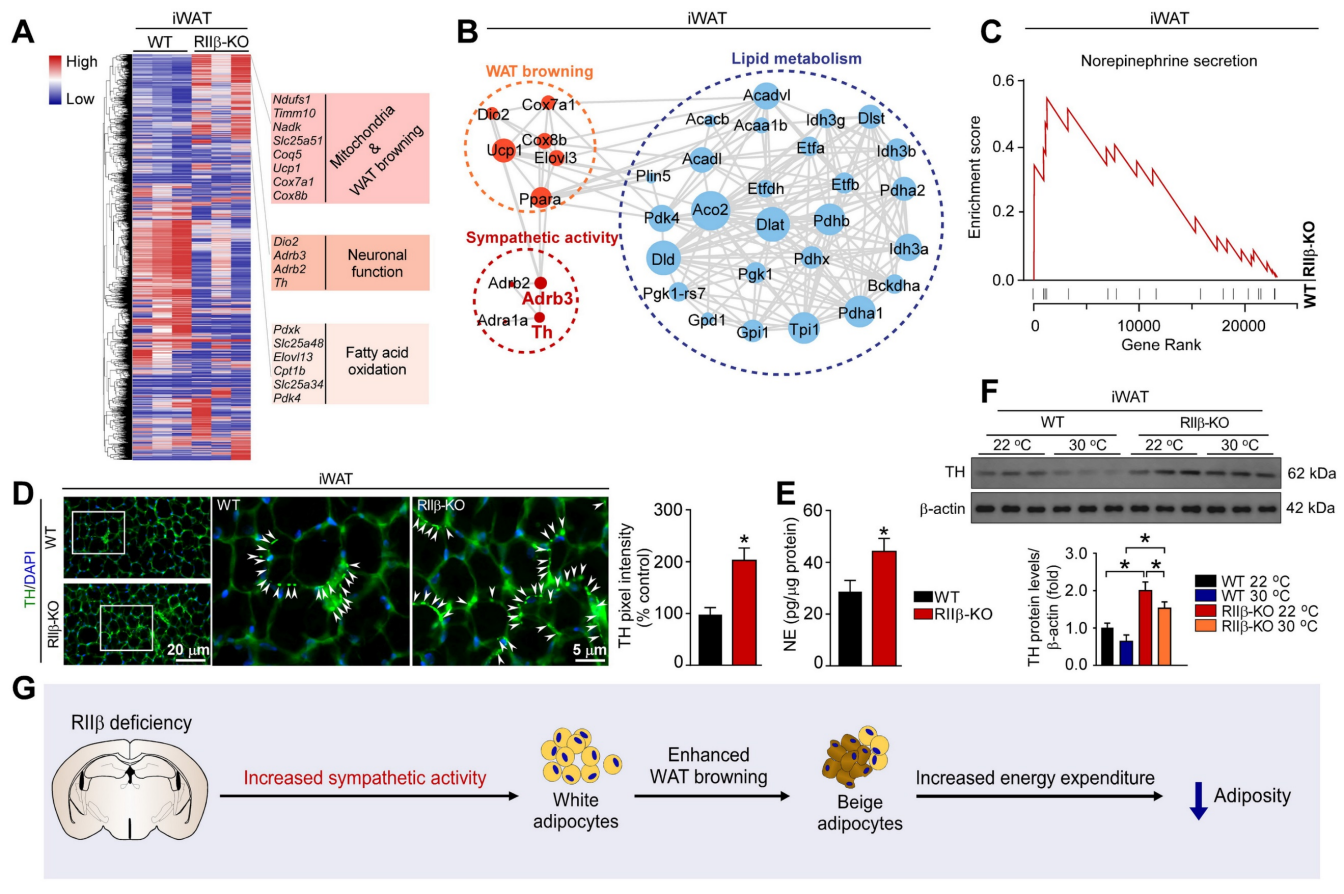
** PKA-RIIβ deficiency elevates WAT sympathetic activity. (A)** Heatmap depicting differentially expressed genes in iWAT. The WAT browning-associated genes in iWAT of WT mice and RIIβ-KO mice are indicated in the heatmap labels. WT n = 3; RIIβ-KO n = 3.** (B)** Analysis of protein-protein interaction networks demonstrates higher expression levels of proteins in RIIβ-KO mice were involved in the regulation of WAT browning, sympathetic activity, and lipid metabolism.** (C)** GSEA shows that the gene set related to NE secretion are significantly upregulated in RIIβ-KO mice.** (D)** Representative immunofluorescence images of tyrosine hydroxylase in iWAT and quantification of the tyrosine hydroxylase. Tyrosine hydroxylase staining is indicated with arrows. Scale bar indicates 20 μm and 5 μm, respectively. **(E)** ELISA of NE content in iWAT. WT 22 °C n = 6; RIIβ-KO 22 °C n = 6.** (F)** Representative immunoblots of TH and β-actin from iWAT, and the quantified ratio of TH/β-actin. P values were determined by two-way ANOVA followed by Tukey's multiple comparisons test. *P < 0.05 and **P < 0.01.** (G)** Proposed mechanism underlying WAT browning of RIIβ-KO mice.

Hypothalamic RIIβ deficiency can induce WAT browning 28. The hypothalamus is a brain region that drives WAT browning via sympathetic nerves 44, 62. However, whether sympathetic nerves are involved in the mediation of RIIβ subunit deficiency-induced WAT browning is undetermined. To explore the mechanism underlying the WAT browning of RIIβ-KO mice, genome-wide transcriptome sequencing analysis was performed to generate a high-resolution transcriptomic profile of iWAT for identifying the differentially expressed genes (DEGs). Gene expression profiles were visualized as a heatmap (Figure 2A). The expression of the genes associated with mitochondria and WAT browning (*Ucp1, Cox7α1, Cox8β*), neuronal function (*Dio2, Adrb3, Adrb2, Th*) and fatty acid oxidation (*Pdxk, Slc25a48, Elovl13, Cpt1b, Slc25a34, Pdk4*), were increased in RIIβ-KO mice (Figure 2A). STRING interaction network also demonstrated that sympathetic activity was closely correlated with WAT browning and lipid metabolism (Figure 2B). By applying GSEA analysis, we found that regulation of norepinephrine (NE) secretion process was remarkably activated in iWAT of RIIβ-KO mice (Figure 2C). The NE levels of iWAT in RIIβ-KO mice were remarkably higher that than of WT mice (Figure 2E). The protein levels of tyrosine hydroxylase (TH), a rate-limiting enzyme in catecholamine synthesis, and also a canonical marker for sympathetic innervation 62, were increased in iWAT of RIIβ-KO mice, as compared with WT control mice (Figure 2D,F). These observations revealed a potential mechanism by which sympathetic nerves may be involved in the mediation of WAT browning of RIIβ-KO mice (Figure 2G). Together, these results revealed an enhanced WAT browning and elevated NE and TH levels in iWAT of RIIβ-KO mice; and also showed that sympathetic nerves may mediate the adipose browning process.

### Sympathetic denervation abrogates PKA-RIIβ deficiency-induced white fat browning

**Figure 3:**
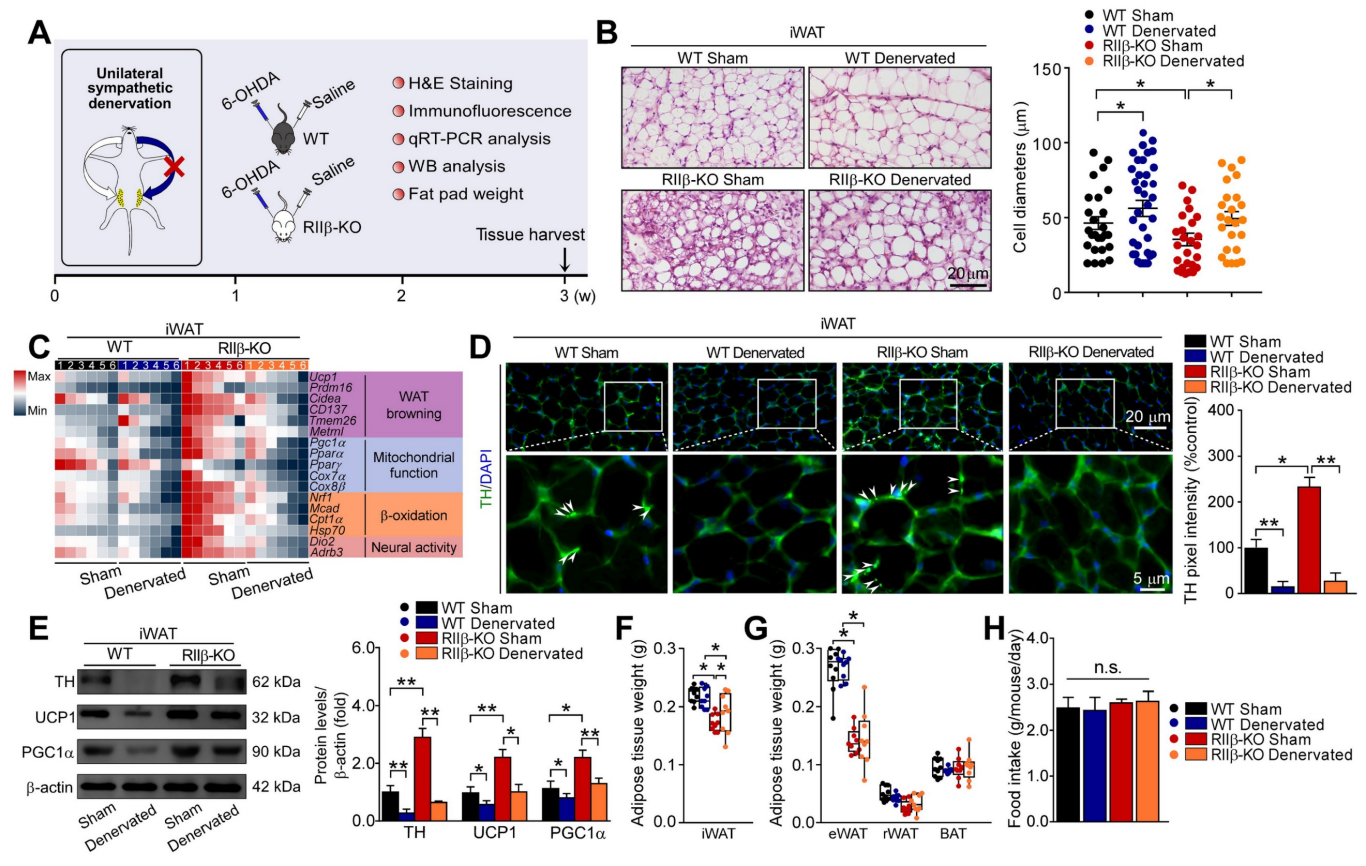
** Sympathetic nerves mediate PKA-regulated WAT browning. (A)** Schematic illustration of experiments. iWAT was unilaterally denervated with 6-OHDA. Tissues were harvested for molecular analyses on day 21. **(B)** Representative images of H&E staining of iWAT and the size profiling of adipocytes from iWAT. Scale bar indicates 20 μm. **(C)** Heatmap shows mRNA levels of browning associated genes in iWAT. WT Sham n = 6; WT Denervated n = 6; RIIβ-KO Sham n = 6; RIIβ-KO Denervated n = 6. **(D)** Representative immunofluorescence images of TH in iWAT. Scale bars indicate 20 μm and 5 μm respectively. **(E)** Representative immunoblots of TH, UCP1, PGC1α and β-actin from iWAT, and the quantified ratio of TH/β-actin, UCP1/β-actin and PGC1α/β-actin. **(F)** Fat-pad weight of iWAT. **(G)** Fat-pad weight of eWAT, rWAT and BAT. **(H)** Food intake. WT Sham n = 9; WT Denervated n = 9; RIIβ-KO Sham n = 9; RIIβ-KO Denervated n = 9. Values show mean ± SEM. P values were determined by two-way ANOVA followed by Tukey's multiple comparisons test. *P < 0.05 and **P < 0.01.

To further determine the role of sympathetic nerves in WAT browning of RIIβ-KO mice, we employed the pharmacologic approach of 6-hydroxydopamine (6-OHDA) to locally denervate the sympathetic fibers in iWAT 62-64. We evaluated whether sympathetic denervation might dampen the WAT browning in RIIβ-KO mice. As shown in Figure 3A, we injected the 6-OHDA unilaterally into the iWAT pad area for chemical denervation, and performed a sham procedure on the contralateral side. H&E staining demonstrated that the denervated iWAT showed larger cell size and cytoplasmic unilocular lipid droplets, whereas the sham-operated contralateral iWAT showed smaller adipocytes containing multilocular lipid droplets (Figure 3B). Unilateral sympathetic denervation attenuated WAT browning and dramatically reduced protein level of TH, as compared with that of the contralateral sham-operated fat pad in RIIβ-KO mice (Figure 3C-E). In WT mice, we found that the denervation also slightly affected the expressions of these markers and cellular morphology in iWAT (Figure 3B-E). Importantly, sympathetic denervation augmented fat-pad weights in RIIβ-KO mice (Figure 3F-G), without affecting food intake (Figure 3H). Taken together, these results indicate that sympathetic neural signals may essentially mediate the WAT browning process in RIIβ-KO mice.

### Deletion of β_3_-adrenergic receptor abolishes PKA-regulated white fat browning

**Figure 4:**
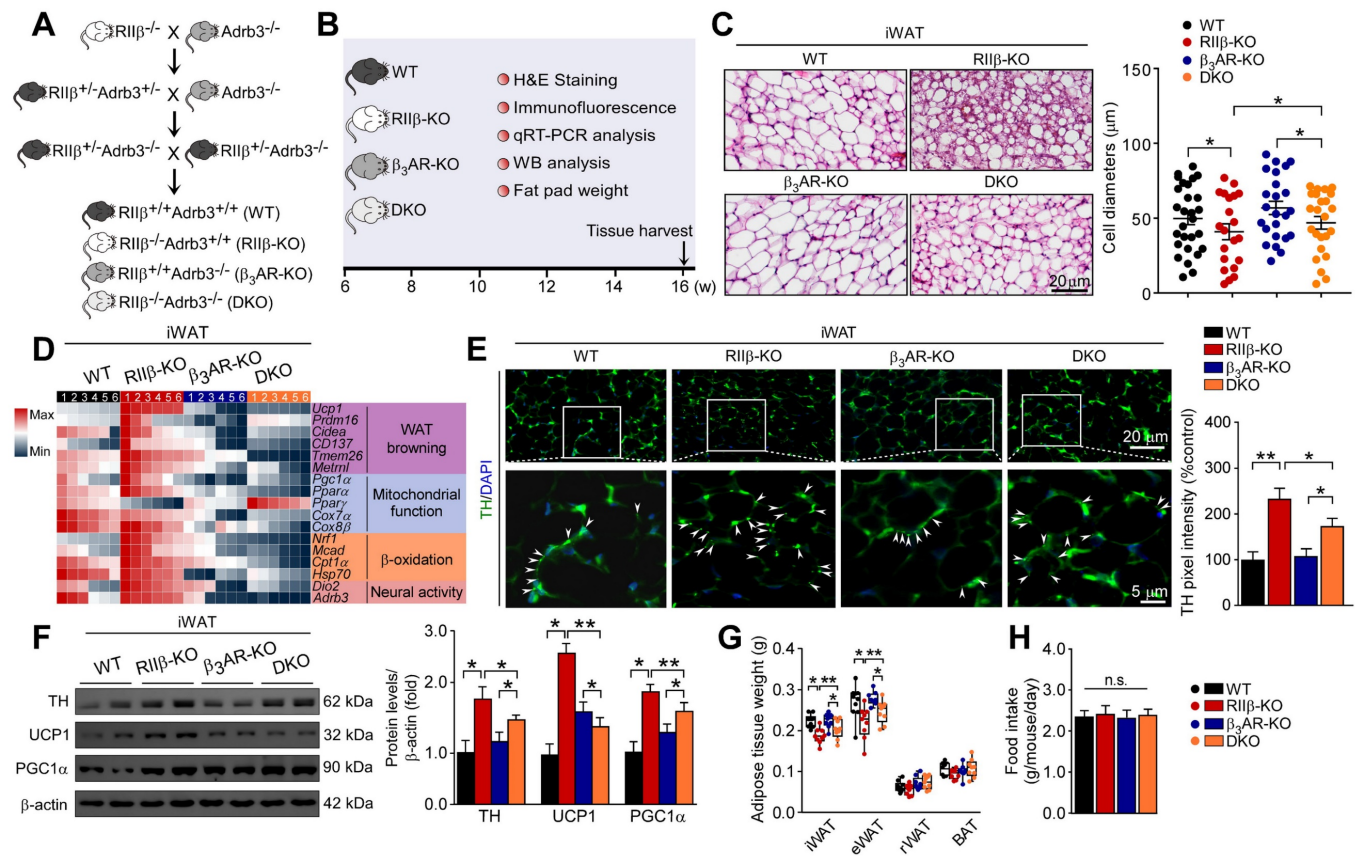
** Deletion of β_3_AR abolishes PKA-RIIβ deficiency-induced WAT browning. (A)** Breeding strategy for generation of RIIβ^-/-^Adrb3^-/-^ mice. **(B)** Schematic illustration of experiments. Tissues were harvested for molecular analyses at week 16. **(C)** Representative images of H&E staining of iWAT and the size profiling of adipocytes from iWAT. Scale bars indicates 20 μm. **(D)** Heatmap shows mRNA levels of browning associated genes in iWAT. WT n = 6; RIIβ-KO n = 6; β_3_AR-KO n = 6; DKO n = 6. **(E)** Representative immunofluorescence images of TH in iWAT. Scale bar indicate 20 μm and 5 μm respectively. **(F)** Representative immunoblots of TH, UCP1, PGC1α and β-actin from iWAT, and the quantified ratio of TH/β-actin, UCP1/β-actin and PGC1α/β-actin. **(G)** Fat-pad weight. **(H)** Food intake. WT n = 9; RIIβ-KO n = 9; β_3_AR-KO n = 9; DKO n = 9. Values show mean ± SEM. P values were determined by two-way ANOVA followed by Tukey's multiple comparisons test. *P < 0.05 and **P < 0.01.

To validate whether the induction of WAT browning could be in a β_3_-adrenergic signaling-dependent manner, we generated RIIβ/Adrb3 double-knockout (DKO) mice (Figure 4A-B). The literature shows that the sympathetic nerve fibers form synapse-like structures to envelop the adipocytes; and the released NE from sympathetic terminals activates the β_3_-adrenergic signal of adipocytes, which is mandatory for the activation of WAT browning 12, 65. We observed that the WAT browning phenotype was absent in the DKO mice, as compared with RIIβ-KO mice. The browning-associated morphological phenotypes of iWAT (Figure 4C), expression levels of genes associated with WAT browning (Figure 4D), and protein levels of TH, UCP1 and PGC1α (Figure 4E-F), decreased weight of iWAT and eWAT (Figure 4G) were normalized in the DKO mice. Remarkably, knockout of adrb3 gene dampened lean phenotypes of RIIβ-KO mice (final weight mean ± SEM: WT, 31.0 ± 0.8 g; RIIβ-KO, 29.1 ± 0.6 g; β_3_AR-KO, 31.2 ± 0.9 g; DKO, 32.1 ± 0.3 g), and did not alter food intake (Figure 4H). Taken together, these findings indicate that sympathetic nerves mediate the adipose browning process of RIIβ-KO mice, confirming the importance of CNS-adipose loop in the regulation of adipose homeostasis.

### SNS-mediated WAT browning underlies PKA-RIIβ deficiency-induced obesity-resistant phenotype

**Figure 5:**
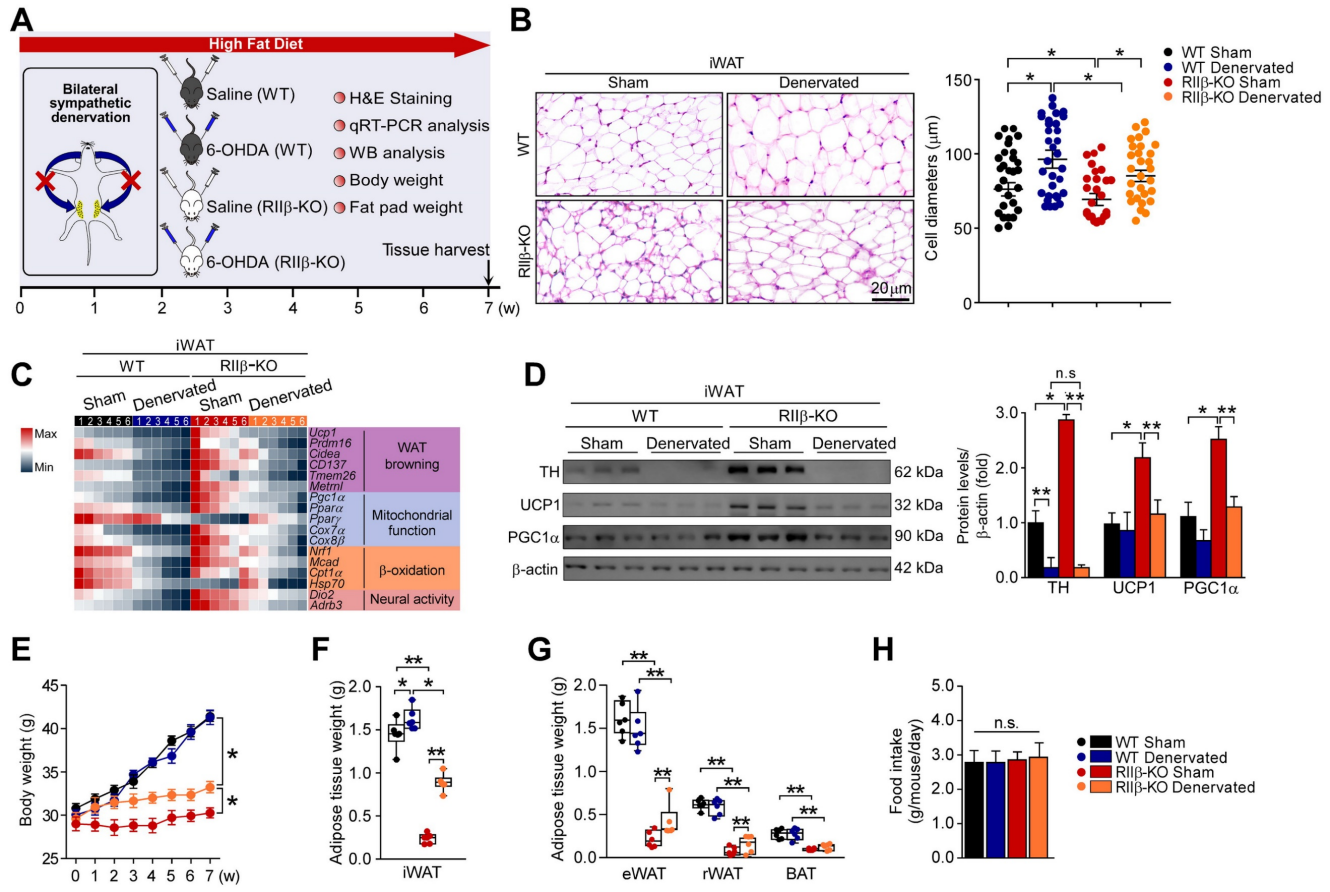
** Sympathetic denervation dampens the WAT browning and DIO-resistant phenotypes. (A)** Schematic illustration of experiments. iWAT was bilaterally denervated with 6-OHDA, mice were kept on HFD for seven weeks. Seven weeks later, tissues were harvested for molecular analyses. **(B)** Representative images of H&E staining of iWAT and the size profiling of adipocytes from iWAT. Scale bar indicates 20 μm. WT Sham n = 9; WT Denervated n = 9; RIIβ-KO Sham n = 9; RIIβ-KO Denervated n = 9. **(C)** Heatmap shows mRNA levels of browning-associated genes in iWAT. WT Sham n = 6; WT Denervated n = 6; RIIβ-KO Sham n = 6; RIIβ-KO Denervated n = 6. **(D)** Representative immunoblots of TH, UCP1, PGC1α and β-actin from iWAT, and the quantified ratio of TH/β-actin, UCP1/β-actin and PGC1α/β-actin. **(E)** Body weight. **(F)** Fat-pad weight of iWAT. **(G)** Fat-pad weight of eWAT, rWAT and BAT. **(H)** Food intake. WT Sham n = 9; WT Denervated n = 9; RIIβ-KO Sham n = 9; RIIβ-KO Denervated n = 9. Values show mean ± SEM. P values were determined by two-way ANOVA followed by Tukey's multiple comparisons test. *P < 0.05 and **P < 0.01.

RIIβ-KO mice are resistant to diet-induced obesity 40, 66. To determine whether WAT browning contributes to the obesity-resistant phenotypes of RIIβ-KO mice, we assessed the impact of sympathetic denervation on high-fat diet-fed (HFD-fed) RIIβ-KO mice. We performed a bilateral sympathetic denervation in iWAT of RIIβ-KO mice fed with HFD (Figure 5A). The decreased WAT browning and reduced activity of SNS was observed in iWAT of these bilaterally denervated RIIβ-KO mice (Figure 5B-D). In parallel, we found that the RIIβ-KO mice received bilateral sympathetic denervation in iWATs gained more body weight (final weight mean ± SEM: WT Sham, 41.2 ± 0.8 g; WT Denervated, 41.4 ± 0.9 g; RIIβ-KO Sham, 30.3 ± 0.6 g; RIIβ-KO Denervated, 33.2 ± 0.7 g) and fat pad weights under HFD challenge, as compared with the sham-operated groups (Figure 5E-G). The food intake was unchanged (Figure 5H). In addition, the bilateral sympathetic denervation did not alter expression levels of WAT browning associated genes, and also did not change body weight and fat pad weights in WT mice (Figure 5B-G). Collectively, these results suggest that SNS-mediated WAT browning underlies the obesity-resistant phenotypes of RIIβ-KO mice.

### Adipocytic mTOR and lipin1 are related to PKA-RIIβ deficiency-induced WAT browning

**Figure 6:**
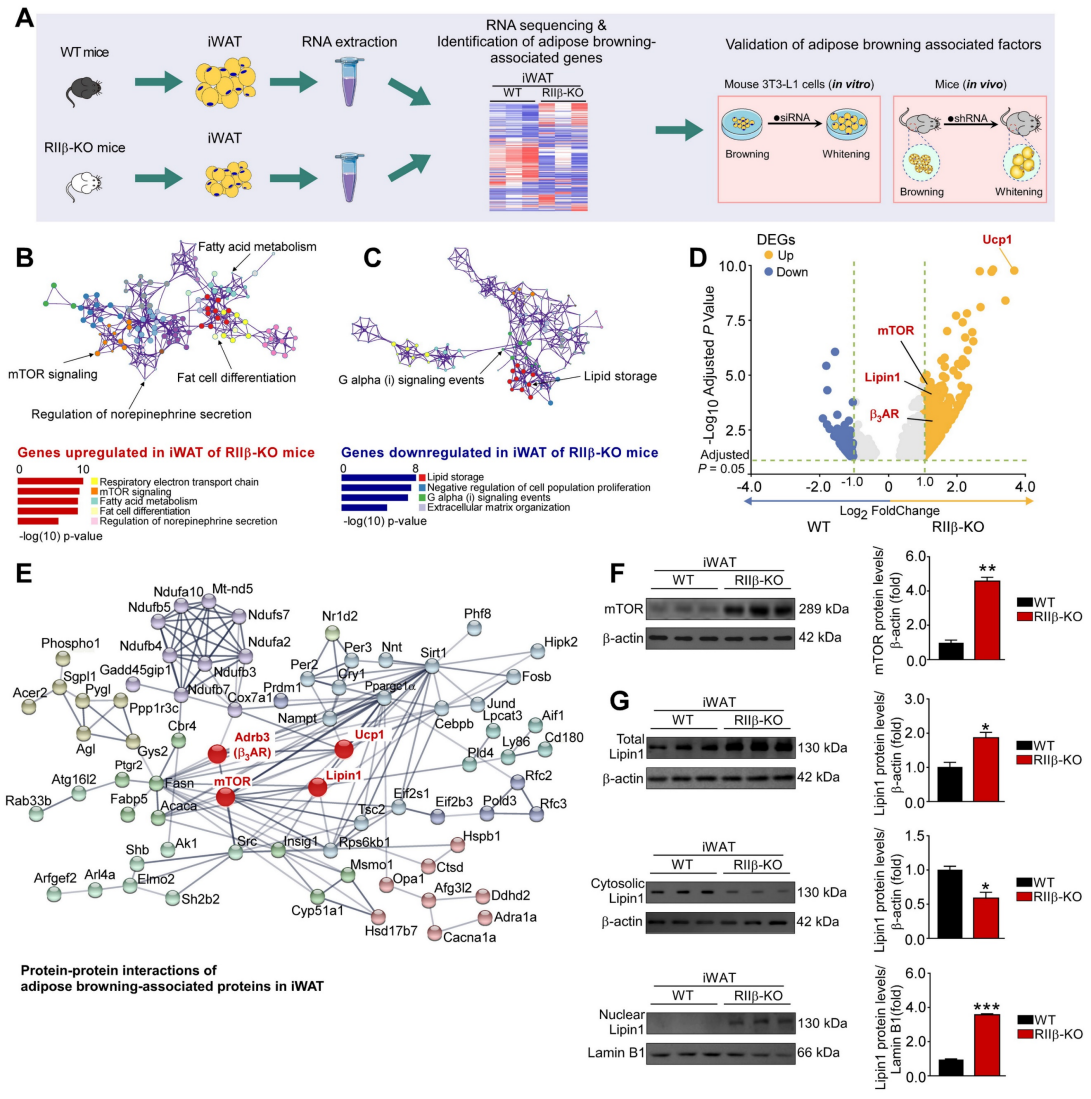
** Adipocytic mTOR and Lipin1 are related to PKA-regulated WAT browning. (A)** Schematic illustration of experiments. **(B, C)** GO analysis is based on DEGs that have a p-value smaller than 0.05. Enrichment analysis for Gene Ontology terms among the genes of a gene-trait correlation module is performed using Metascape. **(D)** Volcano plot displays DEGs of RIIβ-KO mice compared to WT mice. **(E)** Analysis of protein-protein interaction networks demonstrates mTOR and lipin1 may mediate WAT browning. **(F)** Representative immunoblots of mTOR and β-actin, and the quantified ratio of mTOR/β-actin. **(G)** Representative immunoblots of total Lipin1, cytosolic Lipin1, β-actin, nuclear lipin1 and lamin B1 from iWAT, and the quantified ratio of cytosolic lipin1/β-actin and nuclear lipin1/lamin B1. WT n = 6; RIIβ-KO n = 6. Values show mean ± SEM. P values were determined by non-paired two-tailed Student's t-test. *P < 0.05.

To gain insights into the molecular mechanism underlying sympathetic nerve-mediated WAT browning, we carried out an integrative analysis to identify the adipose browning associated factors (Figure 6A). GO (gene ontogeny) and KEGG (Kyoto Encyclopedia of Genes and Genomes) analyses showed that the pathways associated with respiratory electron transport chain, mTOR signaling, fatty acid metabolism, fat cell differentiation and regulation of NE secretion were activated (Figure 6B and Figure S1A-D), whereas activities of the pathways associated with lipid storage and G_i_ signaling events were downregulated in iWAT of RIIβ-KO mice (Figure 6C). We found that the expression levels of mTOR complex 1 (mTORC1)-related genes including mTOR, Rptor, Mlst8, and Rps6kb1 (S6K) were increased in iWAT of RIIβ-KO mice. Whereas, the expression levels of mTOR complex 2 (mTORC2)-related genes were not changed markedly. These findings showed that the mTORC1 pathway was activated in iWAT of RIIβ-KO mice, suggesting the potential relationship between mTORC1 signaling and the WAT browning (Figure S1A-D). To identify the potential molecular factors through which sympathetic nerves mediate WAT browning, we performed the volcano plot analysis for the sequencing data (RIIβ-KO versus WT mice) (Figure 6D). Volcano plots showed that mTOR and lipin1 were markedly upregulated in iWAT of RIIβ-KO mice (Figure 6D), revealing mTOR and lipin1 may be the mediators of WAT browning. Protein-protein network interactions analysis using STRING further predicted a potential interaction and a high clustering coefficient among the adipose browning-associated proteins in iWAT (Figure 6E). This analysis demonstrated that mTOR and lipin1 may closely interact with the genes associated with sympathetic activity (*β_3_AR*), WAT browning (*Ucp1*), and other genes related to mitochondria and fatty acid oxidation. Of note, mTOR plays a critical role in the process of WAT browning 55. Lipin1 is a bifunctional intracellular protein that is emerging as a critical regulator of fat metabolism 58. Lipin1 regulates metabolism by acting as a coregulator of transcriptional factors, and it can translocate from the cytosol to the nucleus to regulate the expression of WAT browning-associated genes 55. We observed a remarkable upregulation in the expression of Lipin1-targeted/regulated genes, including the genes related to WAT browning (UCP1, Pgc1α, Cidea, Cox7α1, Cox8β, Pparα), fatty acid oxidation (Acox1, Cpt1α, Acadvl, Acadm), and fatty acid synthesis (Srebf1, Fasn, Scd1, Mttp), showing that the Lipin1 may be closely involved in the regulation of adipose browning as well as fatty acid synthesis and oxidation in WAT of RIIβ-KO mice (Figure S1E). Moreover, we observed that protein levels of mTOR, total and nuclear lipin1 were increased, whereas protein level of cytosolic lipin1 was decreased in iWAT of RIIβ-KO mice (Figure 6F-G), suggesting a heightened activity of mTOR and lipin1 in iWAT of RIIβ-KO mice. Taken together, these findings suggest that mTOR and lipin1 may act as factors involved in mediating WAT browning in RIIβ-KO mice.

### mTOR and lipin1 are identified as adipocytic mediators of PKA-RIIβ deficiency-induced WAT browning

**Figure 7:**
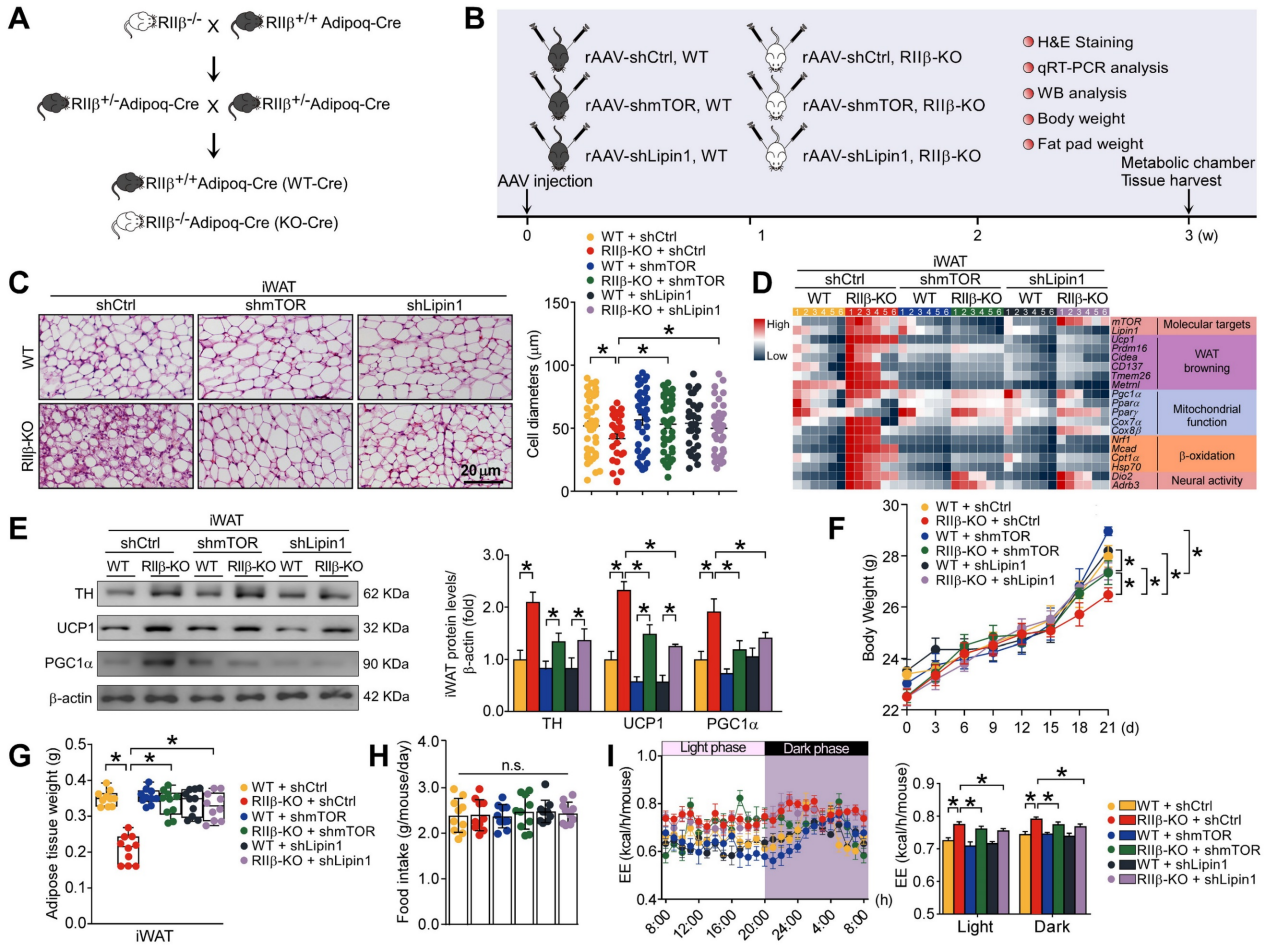
** mTOR and lipin1 are identified as adipocytic mediators of PKA-regulated WAT browning. (A)** Breeding strategy for generation of RIIβ^+/+^Adipoq-Cre mice (WT) and RIIβ^-/-^Adipoq-Cre mice (RIIβ-KO). **(B)** Schematic illustration of experiments. **(C)** Representative images of H&E staining of iWAT and the size profiling of adipocytes from iWAT. Scale bar indicates 20 μm. WT shCtrl n = 9; RIIβ-KO shCtrl n = 9; WT shmTOR n = 9; RIIβ-KO shmTOR n = 9; WT shLipin1 n = 9; RIIβ-KO shLipin1 n = 9. **(D)** Heatmap shows mRNA levels of browning associated genes in iWAT. WT shCtrl n = 6; RIIβ-KO shCtrl n = 6; WT shmTOR n = 6; RIIβ-KO shmTOR n = 6; WT shLipin1 n = 6; RIIβ-KO shLipin1 n = 6. **(E)** Representative immunoblots of TH, UCP1, PGC1α and β-actin from iWAT, and the quantified ratio of TH/β-actin, UCP1/β-actin and PGC1α/β-actin. Values show mean ± SEM. P values were determined by non-paired two-tailed Student's t-test. *P < 0.05. **(F)** Body weight. **(G)** Weight of iWAT. **(H)** Food intake. WT shCtrl n = 10; RIIβ-KO shCtrl n = 10; WT shmTOR n = 10; RIIβ-KO shmTOR n = 10; WT shLipin1 n = 10; RIIβ-KO shLipin1 n = 10. **(I)** Energy expenditure (n = 5 per group). Data are presented as the mean ± SEM. P values were determined by two-way ANOVA followed by Tukey's multiple comparisons test. *P < 0.05.

To determine whether mTOR and lipin1 may mediate the WAT browning of RIIβ-KO mice, we crossed RIIβ-KO mice with adiponectin-Cre mice (Figure 7A) and used adeno-associated viruses (AAVs) expressing short hairpin RNA (shRNA) targeting mTOR or lipin1 to knockdown endogenous mTOR or lipin1 in iWAT (Figure 7B and Figure S2A). We observed that targeted knockdown of mTOR or lipin1 in iWAT diminished the WAT browning phenotypes of RIIβ-KO mice (Figure 7C), and normalized the levels of markers associated with WAT browning, body weight (final weight mean ± SEM: WT shCtrl, 28.4 ± 0.3 g; WT shmTOR, 29.0 ± 0.2 g; WT shLipin1, 28.6 ± 0.3 g; RIIβ-KO shCtrl, 26.4 ± 0.3 g; RIIβ-KO shmTOR, 27.7 ± 0.3 g; RIIβ-KO shLipin1, 27.8 ± 0.4 g) and fat pad weights (Figure 7D-G). The body weight and adiposity of WT mice were unchanged upon mTOR or lipin1 knockdown; while the body weight and adiposity of RIIβ-KO mice were significantly increased after mTOR or lipin1 knockdown. Under the condition of mTOR or lipin1 knockdown, the body weight and adiposity were increased in RIIβ-KO mice; whereas the body weight and adiposity were unchanged in WT mice (Figure 7 F-G and Figure S2B). No difference in the food intake was observed among all groups (Figure 7H). On the other hand, the increased EE of RIIβ-KO mice was also reduced by knockdown of mTOR or lipin1 in iWAT (Figure 7I). These results suggest the adipocytic mTOR and lipin1 mediate WAT browning in RIIβ-KO mice.

### Knockdown of mTOR and lipin1 diminishes WAT browning-related responses

**Figure 8:**
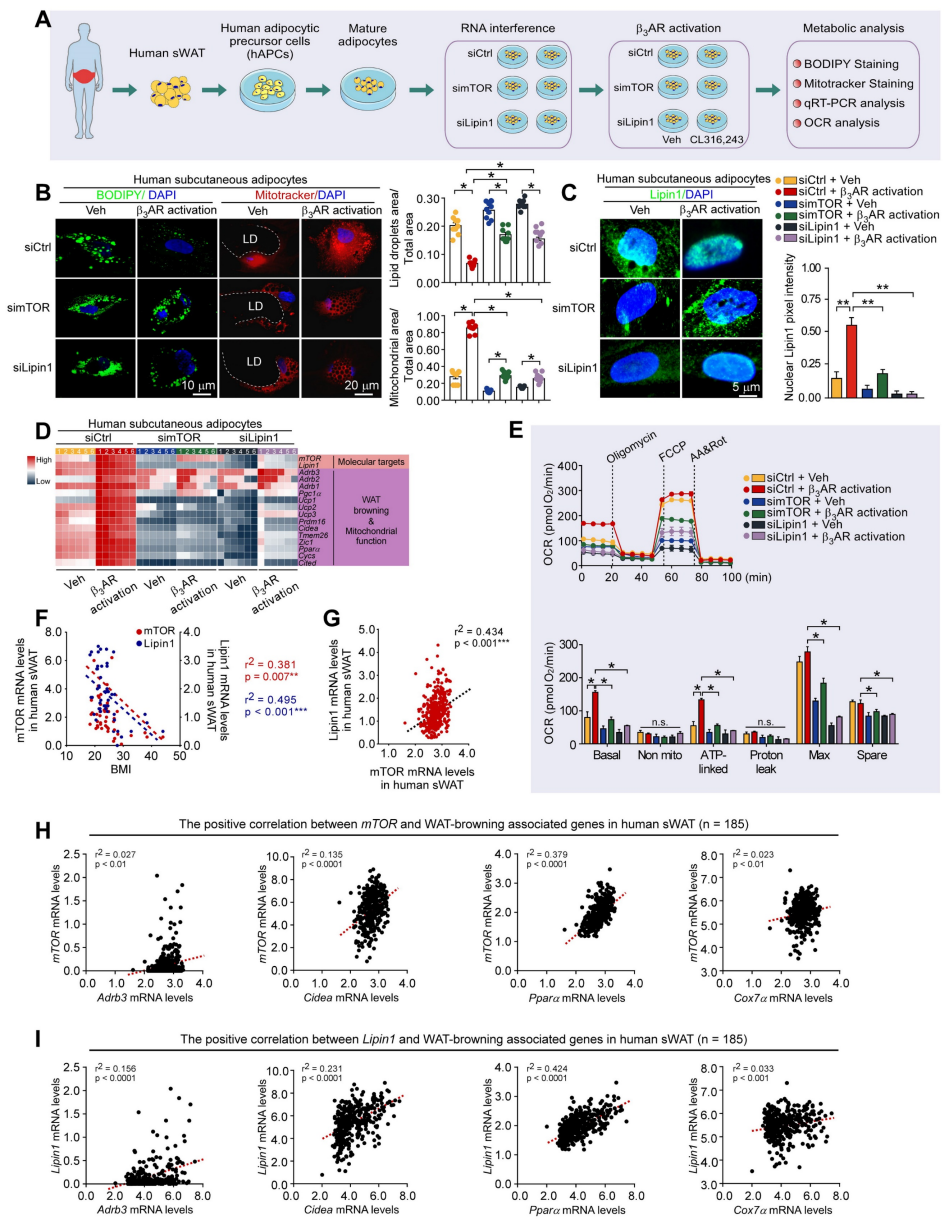
** mTOR and lipin1 mediate β-adrenergic stimulation-induced WAT browning-related responses in human adipocytes. (A)** Schematic illustration of experiments. **(B)** Representative immunofluorescence images of BODIPY (green) and Mitotracker (red) of human subcutaneous adipocytes, and the quantified ratio of lipid droplets area/total area and mitochondrial area/total area. Values show mean ± SEM. P values were determined by two-way ANOVA followed by Tukey's multiple comparisons test. *P < 0.05. **(C)** Representative immunofluorescence images of lipin1 in human subcutaneous adipocytes, and quantified nuclear lipin1 pixel intensity. siCtrl Veh n = 6; siCtrl β_3_AR activation n = 6; simTOR Veh n = 6; simTOR β_3_AR activation n = 6; siLipin1 Veh n = 6; siLipin1 β_3_AR activation n = 6. P values were determined by two-way ANOVA followed by Tukey's multiple comparisons test. *P < 0.05. **(D)** Heatmap shows mRNA levels of WAT browning associated genes in human subcutaneous adipocytes. siCtrl Veh n = 6; siCtrl β_3_AR activation n = 6; simTOR Veh n = 6; simTOR β_3_AR activation n = 6; siLipin1 Veh n = 6; siLipin1 β_3_AR activation n = 6. **(E)** Cellular respirometry of mTOR or lipin1 knockdown human adipocytes. Time course and oxygen consumption rate (OCR) were recorded by microplate-based respirometry (Seahorse XF96 Analyzer) under basal conditions and during successive injection of 5 μM oligomycin, 0.5 μM isoproterenol, 1 μM FCCP, and 5 μM antimycin A. n = 6 wells for control and 6-8 wells per treatment. Values show mean ± SEM. P values were determined by two-way ANOVA followed by Tukey's multiple comparisons test. *P < 0.05. **(F)** Correlation between mTOR and lipin1 expression levels in abdominal sWAT and BMI in humans. mTOR and lipin1 mRNA expression levels were quantified by qPCR and normalized to β-actin mRNA. Statistical analysis was performed by Pearson correlation. **(G)** Pearson's r correlations for mTOR and lipin1 with WAT-browning associated genes in human sWAT. **(H, I)** Pearson's r correlations for lipin1 with mTOR in human WAT. For analysis, we used RNA sequencing data from the Genotype-Tissue Expression project (archived at http://www.genenetwork.org).

To validate whether mTOR and lipin1 may mediate WAT browning induced by sympathetic nerves, the human adipocytes derived from human adipocytic precursor cells (hAPCs) isolated from human adipose tissue as well as the mouse 3T3-L1 adipocytes were used; and gene knockdown and pharmacological approaches were exploited (Figure 8A). The small interfering RNA (siRNA)-mediated gene knockdown approach was utilized to deplete endogenous mTOR and lipin1 in adipocytes; and the selective β_3_ adrenergic receptor agonist CL316,243 was used to treat adipocytes for simulating sympathetic activation (Figure 8A) 67, 68. Representative BODIPY and MitoTracker staining were shown in Figure 8B and Figure S3A. mTOR and lipin1 knockdown by siRNA inhibited adipocytic lipolysis and decreased mitochondrial activity in both human and mouse adipocytes (Figure 8B, D and Figure S3A, C). We observed that β_3_AR activation caused a remarkable nuclear translocation of lipin1, while this could be suppressed by mTOR and lipin1 knockdown in both human and mouse adipocytes (Figure 8C and Figure S3B). Further, we found that elevated expression levels of WAT browning markers induced by CL316,243 were dampened in the condition of mTOR or lipin1 knockdown (Figure 8D and Figure S3C). Under the condition of β_3_AR activation, the adipocyte-specific knockdown of mTOR decreased lipin1 expression level, whereas knockdown of lipin1 did not affect mTOR expression level, suggesting that mTOR may regulate lipin1 function, and lipin1 may act downstream of mTOR (Figure S3D).

Further, to analyze the functional roles of mTOR and lipin1 in mediating mitochondrial activity and energy expenditure, the measurement of oxygen consumption rate (OCR) was performed. CL316,243 treatment increased basal, ATP-linked and maximum respiratory capacity in human adipocytes (Figure 8E). There were no differences in OCR for non-mitochondrial and proton leak (Figure 8E). Of note, OCR for basal, ATP-linked, maximal respiration and spare respiratory capacity was downregulated after knockdown of mTOR or lipin1 (Figure 8E), suggesting the importance of mTOR and lipin1 in the regulation of oxidative respiration and energy expenditure. Collectively, these results validate that mTOR and lipin1 may be critical for mediating the energy expenditure induced by sympathetic activation in adipocytes.

### Adipocytic mTOR and lipin1 may serve as targets for novel anti-obesity therapies

To provide a comprehensive understanding of the role of mTOR and lipin1 in obesity, we recruited metabolically healthy volunteers. Based on the eligibility criteria 69, a total of 47 participants were included in this study (Figure S4). We observed that the expression levels of mTOR and lipin1 in human sWAT were negatively correlated with BMI (Figure 8F). By using public datasets, we also observed that mRNA levels of mTOR and Lipin1 were positively correlated with BMI (Figure S5), validating the important roles of mTOR and Lipin1 in regulating adipose tissue browning.

**Figure 9:**
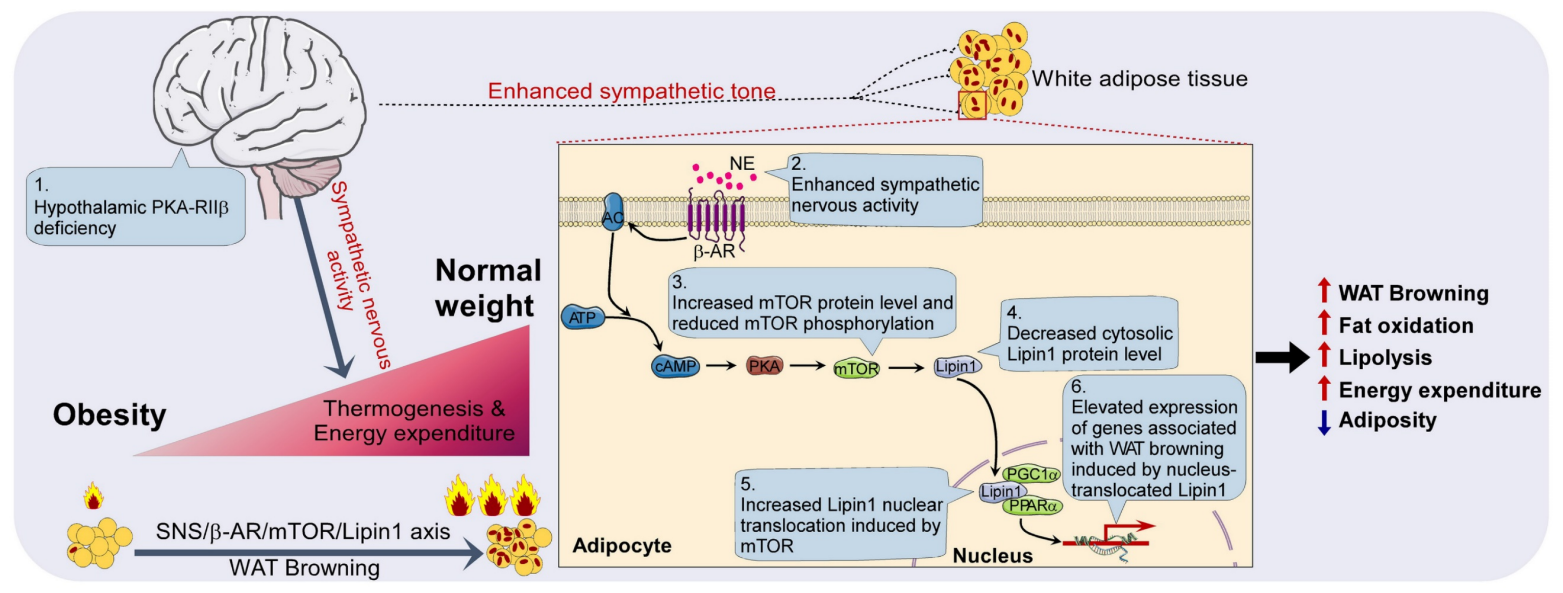
Diagram illustrating the peripheral mechanism by which mTOR and Lipin1 mediate PKA-regulated WAT browning.

Moreover, an independent human database, genotype-tissue expression project (archived at http://www.genenetwork.org) 70, validated that lipin1 expression level in human abdominal sWAT was positively correlated with mTOR (Figure 8G). In human adipose tissue, expression levels of mTOR and Lipin1 exhibited a positive correlation with Cidea, Pparα, Adrb3 and Cox7α (Figure 8H-I). Taken together, these findings suggest that mTOR/lipin1 signaling may be a critical downstream pathway of βAR participating in the regulation of WAT browning, showing novel roles for mTOR and lipin1 in the regulation of energy metabolism; β_3_-activated mTOR-lipin1 axis may critically underlie the molecular basis of PKA-regulated WAT browning, providing adipose target candidates for the development of drugs to treat obesity (Figure 9).

## Discussion

The prevalence of obesity has increased worldwide, reaching pandemic levels 5, 71. Currently, there is no established prevention or viable long-term treatment strategies for obesity 72. Therefore, a better understanding of the mechanism underlying the regulation of adipose homeostasis may help discover the effective molecular targets for combating obesity. The experimental studies using model mice are critical to address these issues 73, 74. In this regard, the RIIβ-KO mouse line has become one of the important models, owing to RIIβ-KO mice display significant lean and robust WAT browning phenotypes and are resistant to diet-induced obesity 27, 28, 39, 41, 66. Nevertheless, the molecular mechanisms behind these phenotypes remain to be determined.

It was reported that the eWAT of RIIβ-KO mice has increased baseline lipolysis activity but shows blunted lipolytic response after β-adrenergic stimulations 39, 75. This may be attributed to the predominance of the R1α subunit in the condition of RIIβ deficiency, which also leads to an enzymatic subtype-switch from Type-II PKA to Type-I PKA. In adipocytes, Type-I PKA is more sensitive to sympathetically-derived norepinephrine 39, and thus the basal lipolysis levels in WAT of RIIβ-KO mice were higher than that of WT mice. Notably, owing to the absence of the RIIβ subunit, there is a net decrease of ~50% in R subunits; therefore, when exposed to strong exogenous β-adrenergic stimulation, such as isoproterenol (0.3 mg/kg) or CL316,243 (1.0 mg/kg), the loss of total R subunits results in a reduced peak PKA activity, which causes a blunted lipolytic response to exogenous β-adrenergic stimulation in white adipocytes of RIIβ-KO mice, as compared to that of WT mice^39^, ^75^. In addition, it is noteworthy that under the condition of RIIβ deficiency, the expression levels of the WAT browning-associated genes such as Ucp1, Prdm16, Pgc1α, CD137, Tbx1, Cidea, and Elovl3 in primary iWAT adipocytes of RIIβ-KO mice are higher than those in WT 42, showing that the RIIβ deficiency does not affect the differentiation and thermogenic parameters of white adipocytes.

It has been shown that RIIβ re-expression using adipocyte-specific Cre does not affect the lean phenotype of RIIβ-KO mice; while RIIβ reexpression in hypothalamic GABAergic neurons rescues the phenotype 28, showing the importance of hypothalamic RIIβ-PKA in the regulation of WAT browning.

In this study, our findings revealed that the absence of RIIβ gene leads to elevated sympathetic activity, causing WAT browning and lowered adiposity at room temperature or thermoneutrality, although the thermoneutrality diminished the RIIβ deficiency-induced WAT browning and weight loss. These results determined the existence of WAT browning in the RIIβ-KO mice, demonstrating that the WAT browning may underlie the anti-obesity phenotypes of RIIβ-KO mice.

Moreover, the genetic knockout of adrb3 or pharmacological sympathetic denervation abrogates the WAT browning. These findings demonstrate that SNS essentially mediates WAT browning of RIIβ-KO mice; and the increased activity of sympathetic nerve may be one of the driving forces for generating WAT browning.

Identification of the adipocytic factors that are involved in mediating WAT browning can be helpful in finding molecular targets for treatment of obesity 76-81. It is well recognized that the βAR/cAMP/PKA/HSL pathway is a key signaling that controls lipolysis in mammals 82, 83. However, the intracellular signaling pathways that mediate WAT browning remain poorly understood. In this study, by analyzing the adipose samples from RIIβ-KO mice, we identified that the genes encoding mTOR and lipin1 are particularly activated in the browned WAT. mTOR is a key hub for coordination of both catabolic and anabolic processes of cells 49. For example, mTOR is required for the WAT browning induced by βAR activation 55, whereas it also mediates insulin-induced lipid storage and adipose expansion 84. Activation of mTOR induces lipid catabolism via mitochondrial biogenesis 56, 85, 86. Inhibition of mTOR by rapamycin, a potent inhibitor of mTOR, robustly reduces thermogenic gene expression in WAT and decreases UCP1 expression in 3T3-L1 adipocytes 87. mTOR is also a downstream target of βAR signaling in white adipocytes 55. Activation of mTORC1 is essential for development of UCP1-containing beige cells in WAT 55. These observations imply that mTOR may be critical for mediating β_3_-adrenergic signal-induced WAT browning. In pathological conditions or upon genetic mutations, an increment of mTOR total protein levels can be observed, showing its key roles as a critical regulator in the cellular response to endogenous or exogenous stimuli 50-52. In this study, we tested the functional roles of mTOR in the process of WAT browning, and our findings show that the protein levels of mTOR was increased, and adipocyte-specific knockdown of mTOR dampens WAT browning in RIIβ-KO mice. Collectively, these findings indicate that mTOR may be a critical downstream pathway of β_3_AR for mediating WAT browning.

Lipin1 is highly expressed in adipocytes 88, 89. In the cell nucleus, lipin1 serves as a co-regulator of DNA-binding transcription factors to promote lipid metabolism 56. In the cytosol, lipin1 acts as a phosphatidic acid phosphatase (PAP), and it may account for all of the PAP activity in WAT 56, 57. The cell nucleus-localized lipin1 interacts with PPARα and PGC1α to modulate fatty acid oxidation 89, 90. Lipin1 level is positively correlated with PPARα gene expression and insulin sensitivity in WAT 88. The loss-of-function of lipin1 leads to manifest lipodystrophy and insulin resistance 90. Lipin1 knockdown dampens the differentiation of preadipocytes, whereas lipin1 overexpression enhances the differentiation 91-93. These observations suggest that the function of lipin1 in cell nucleus is critical for the regulation of adipocyte differentiation and lipid metabolism. In the present study, we found that the cell nuclear entry of lipin1 is increased in iWAT of RIIβ-KO mice, and adipocyte-specific knockdown of lipin1 weakens the WAT browning. Taken together, we suggest that lipin1 may positively regulate WAT browning, and this may contribute to the lipid catabolic state of RIIβ-KO mice.

Emerging evidence shows that mTOR is upstream signaling component that regulates lipin1 activity 56. Lipin1 may act as a target of mTORC1; and mTORC1 may regulate the function of lipin1 by controlling its nuclear entry 56. In this study, we found that knockdown of mTOR or lipin1 in adipocytes dampens the WAT browning of RIIβ-KO mice, demonstrating a crucial role of mTOR/lipin1 axis in the regulation of WAT browning.

Our research aligns with the ongoing exploration of GPCR signal diversity in the regulation of lipid metabolism mediated by the SNS. The nuanced specificity and variety of GPCR interactions with their ligands, combined with distinct downstream signaling pathways, outline a highly complex regulatory network 16-18. This intricacy allows the SNS to adapt dynamically to fluctuating metabolic demands and environmental stimuli 7, 21. Within this framework, the roles of mTOR and Lipin1 as key mediators in adipocyte biology and lipid metabolism may become even more pertinent. The interplay of these factors with G protein-coupled receptors, such as the βAR at downstream of the SNS, opens up fascinating research opportunities 15, 23. In this study, we suggest that exploring the interactions between adipocytic βAR pathways and other regulators, including mTOR and Lipin1, is likely to yield novel insights into the mechanism underlying the integrated regulation of WAT browning. Such a comprehensive understanding is vital for the development of targeted therapeutic strategies in obesity and other metabolic disorders.

It was previously reported that RIIβ-KO mice exhibit a robust WAT browning 28. RIIβ reexpression in dorsomedial hypothalamic (DMH) GABAergic neurons abrogates the WAT browning. The activation of DMH GABAergic neurons leads to WAT browning and weight loss. These previous observations show an important role of RIIβ-PKA in regulating WAT browning and metabolism 28. In the present study, we explored the peripheral mechanism underlying WAT browning; and we found that adipocytic βAR-mTOR-Lipin1 pathway mediates the browning of RIIβ-KO mice. Moreover, both human and mouse data show that mTOR and Lipin1 may play key roles in the function related to lipid-metabolism and WAT browning. Together, these studies enrich our understanding of the peripheral mechanism of WAT browning, and provide a new molecular perspective on the roles of βAR-mTOR-Lipin1 axis in prevention or treatment of obesity and related metabolic disorders.

Altogether, these findings indicate that the mTOR/lipin1 axis is essential for WAT browning, highlighting a novel mechanism of WAT browning regulation, demonstrating novel roles for mTOR and lipin1 in the regulation of energy metabolism. βAR-mTOR-lipin1 axis may underlie the molecular mechanism of PKA-regulated WAT browning, and providing promising adipocyte-specific targets for development of novel anti-obesity therapies.

## Materials and Methods

### Mice

WT mice were acquired from the Department of Laboratory Animal Science of Peking University Health Science Center, as well as from Charles River Laboratories Beijing Branch (Beijing Vital River Laboratory Animal Technology Co., Ltd.). RIIβ^-/-^ (RIIβ-KO) mice were generated and characterized as described previously. RIIβ^-/-^Adrb3^-/-^ mice were generated by crossing the RIIβ-KO mice with Adrb3-KO mice kindly provided by Dr. Wenwen Zeng (Tsinghua University). The RIIβ-KO Adipoq-Cre mice were generated by crossing the RIIβ-KO mice with Adipoq-Cre mice kindly provided by Dr. Weizhen Zhang (Peking University). All animals were matched for both sex and age, with littermates being utilized, as highlighted in the illustrations. The assignment of animals to specific experimental groups was carried out based on their respective genotypes. Mice were subjected to standard chow (Jiangsu Xietong Pharmaceutical Bio-engineering Co., Ltd. #1010010) or a high-fat diet (Research Diets #D12492) and provided unrestricted access to water. The mice were accommodated at temperatures of either 22 - 24 °C or 30 ± 1 °C (considered thermoneutrality), operating on a 12-hour light/dark schedule. For investigations involving food intake and energy expenditure post sympathetic denervation or AAV injection, mice were individually housed. Otherwise, mice were group-housed (two to five animals per cage). Throughout all experimental procedures, efforts were made to minimize the quantity of animals used and to mitigate any potential suffering caused by treatments. All protocols were granted approval by the Institutional Care and Use Committee of Peking University Health Science Center (LA2019340).

### Human subjects

This study was approved by the Ethical Committee of Peking University People's Hospital (2019PHB205-01). Informed consent was obtained from all patients or their parents/ guardians. All data were kept confidential and processed anonymously. The research encompassed 47 samples of human subcutaneous adipose tissue, procured from 6 obese individuals (BMI ≥ 28 kg/m²) and 41 nonobese individuals (18 < BMI < 28 kg/m²), with matching age and sex. These samples were gathered during bariatric surgery or abdominal surgical procedures for benign conditions. The participants, apart from the surgical requirement, displayed apparent health, without any record of excessive alcohol consumption. Notably, none of the patients exhibited evident diabetes or lipodystrophy, and none were undergoing β-blocker-based antihypertensive treatment. Weight was measured before surgery.

### H&E staining

After euthanizing the animals, their adipose tissues were promptly extracted and immersed in a 4% paraformaldehyde solution for 48 hours. Following this fixation, the tissues were subjected to cryopreservation by immersing them in a 25% sucrose solution (weight/volume) overnight, and subsequently frozen within OCT compound (Tissue-Tek). To preserve the samples, they were stored using optimal cutting temperature compound (OCT) for freezing. The preserved samples were then segmented into sections, which were subsequently stained using H&E staining techniques. The assessment of cell size was conducted through the utilization of Image J software 94. Specifically, the measurement of adipocyte size was conducted through the following steps:

Sample Preparation: iWAT samples taken from the mice were fixed and then embedded in paraffin using conventional techniques. The sections were cut at a thickness of 10 microns.

Staining: Sections were stained with Hematoxylin and Eosin to clearly delineate the contours and structures of adipocytes.

Microscopic Imaging: Stained sections were imaged using an optical microscope, capturing detailed images of adipocytes at 40x magnification.

Image Analysis: ImageJ software was employed for image analysis, either manually or automatically outlining the boundaries of adipocytes. For each sample, at least three different fields of view were randomly selected, the area of all visible adipocytes within these fields was calculated, and their average was taken to represent the average size of adipocytes in that sample.

Statistical Analysis: Data collected were subjected to statistical analysis to compare differences in adipocyte size across experimental groups.

### Total protein extraction and western blotting

Proteins were isolated from inguinal white adipose tissue (iWAT) using a RIPA lysis buffer composed of 0.5% NP-40, 0.1% sodium deoxycholate, 150 mM NaCl, and 50 mM Tris-HCl (pH 7.4). This buffer was supplemented with phosphatase inhibitors (B15002, Bimake) and a protease inhibitor cocktail (B14002, Bimake). The tissue was homogenized for five minutes and the resulting lysates were then subjected to centrifugation at 12000 g for 15 minutes at 4 °C. The supernatants collected from the adipose tissue served as the protein extracts.

The protein concentration of each sample was determined using the BCA method. Subsequently, an equivalent amount of protein from each sample was mixed with protein loading buffer, including 5% β-mercaptoethanol (vol/vol), and heated by boiling at 100 ºC for 5 minutes. The proteins were then separated on a 10% SDS-PAGE gel and subsequently transferred to nitrocellulose (NC) membranes.

After blocking the NC membranes for two hours using 5% skim milk, the membranes were exposed to primary antibodies at a 1:1000 dilution in 5% BSA-TSBT at 4 °C overnight. Following this incubation, the membranes were washed thrice with TBST for 15 minutes each and then subjected to a two-hour incubation with secondary antibodies at a 1:5000 dilution in TBST supplemented with 5% skim milk at room temperature. Following three sets of 15-minute washes with 1x TBST, the membranes were subjected to chemiluminescence detection. The intensity of the protein bands was quantified utilizing ImageJ software. The antibodies used in this study are detailed in Table S1. The full immunoblots relating to Figure 1-6 are available in Figures S6-S7.

### Quantitative real-time PCR

Total RNA for quantitative real-time PCR (qPCR) was extracted from tissues with TRIzol (TransGen). The RNA quality and quantity were determined using a NanoDrop 5500 (Thermo). The total RNA was used for mRNA-Sequencing (Novogene, Beijing, China) or qPCR. Total RNA (1 μg) was reverse transcribed to complementary DNA (cDNA) using First Strand cDNA Synthesis Kit, according to the manufacturers' instructions. Quantitative PCR (qPCR) was employed to evaluate the relative expression of mRNAs, utilizing the SYBR Green PCR system manufactured by BioRad. The calculation of the relative expression for the genes of interest was carried out using the comparative Ct method, with GAPDH, β-actin or 18S serving as the internal control for normalization. The Primers used in this study are detailed in Table S2.

### Metabolic chamber

The mice were introduced into metabolic chambers, where they were supplied with fresh food and water daily for a 24-hour acclimation period. On the seventh day, prior to the onset of the dark cycle, we initiated the monitoring of various metabolic parameters over a 24-hour period. This included measuring parameters such as oxygen consumption (VO_2_), carbon dioxide production (VCO_2_), respiratory exchange ratio (RER), energy expenditure (EE), and motor activity. The equipment employed for these measurements included the LE1305 Physiocage 00, LE405 O_2_/CO_2_ Analyzer, and LE400 Air Supply and Switching systems. The collected data was subsequently analyzed using the Metabolism v2.2.01 software. Further, to account for variations in mouse adiposity, adjustments were made to the recorded metabolic parameters.

### Sympathetic denervation of iWAT

8-week-old mice were subjected to a regimen involving 20 microinjections of 6-hydroxydopamine [6-OHDA (Sigma)] at a volume of 1 μl per injection, with a concentration of 9 mg/ml, and dissolved in a solution of 0.15 M NaCl containing 1% (w/v) ascorbic acid. These injections were administered to the right inguinal fat pad or both inguinal fat pads, as described in reference 62. As a control, sham-operated fat pads were treated with an equivalent volume of the vehicle solution. The weights of the mice were carefully monitored throughout the entire experimental period.

Following a period of two weeks (for unilateral injections) or seven weeks (for bilateral injections) subsequent to the administration of 6-OHDA injections, the respective fat pads were collected. These harvested fat pads were then subjected to histological and immunofluorescence assessments, or they were processed for qPCR analysis. Notably, this study did not observe any signs of cardiovascular or renal toxicity related to the experimental procedures.

### Immunofluorescence

Mice samples were swiftly dissected and immersed in a 4% paraformaldehyde solution for a 48-hour fixation period. Subsequently, the tissues were embedded in OCT compound, and sections ranging from 10 to 25 μm were meticulously prepared from the entire tissue block, following established protocols. For the immunofluorescence analysis of tyrosine hydroxylase (TH), the frozen tissue sections were employed.

These sections were initially subjected to a blocking step using 10% (v/v) normal horse serum dissolved in 1X phosphate buffer. They were then subjected to an overnight incubation (at 4 °C) with anti-TH antibody at a dilution of 1:1000. Following a triple wash with PBS for 15 minutes each, the sections were exposed to Alexa-Fluor 488-conjugated secondary antibodies at a dilution of 1:500 for a 2-hour period at room temperature. The nuclei within the sections were counterstained using 4',6-diamidino-2-phenylindole (DAPI). To analyze the stained slides, a microscope (Olympus) was employed at the specified magnification, with images captured utilizing a digital camera.

Quantification of TH fluorescence intensity was performed by using the ImageJ software (http://imagej.nih.gov/ij/). Identical conditions and settings were used for picture acquisition and analysis. A threshold (20 pixels) was set for each image to eliminate background and to create a binary mode image for the quantification of TH staining.

### RNA-sequencing

For the preparation of RNA samples, a total of 3 μg RNA per sample was utilized. The NEBNext® Ultra™ RNA Library Prep Kit for Illumina® (NEB, USA) was employed to generate sequencing libraries according to the manufacturer's instructions, with unique index codes incorporated to identify each sample. Briefly: mRNA was isolated from total RNA using poly-T oligo-attached magnetic beads. Fragmentation of mRNA was conducted using divalent cations under elevated temperature in NEBNext First Strand Synthesis Reaction Buffer (5X). First strand cDNA was synthesized using random hexamer primers and M-MuLV Reverse Transcriptase (RNase H). Second strand cDNA synthesis was carried out using DNA Polymerase I and RNase H. Blunt ends were created by converting remaining overhangs using exonuclease/polymerase activities. Adenylation of 3' ends of DNA fragments was performed, followed by ligation of NEBNext Adaptor with a hairpin loop structure. To select cDNA fragments of preferred lengths (150~200 bp), library fragments were purified using the AMPure XP system. A 3 μl USER Enzyme (NEB, USA) treatment was applied to size-selected, adaptor-ligated cDNA, followed by PCR. PCR amplification was carried out using Phusion High-Fidelity DNA polymerase, Universal PCR primers, and Index (X) Primer. Purification of PCR products was performed using the AMPure XP system, and library quality was evaluated using the Agilent Bioanalyzer 2100 system. Index-coded samples were clustered using the TruSeq PE Cluster Kit v3-cBot-HS (Illumina) on a cBot Cluster Generation System, following the manufacturer's guidelines. Subsequently, the library preparations were subjected to sequencing on an Illumina Hiseq 2000/2500 platform, generating 100 bp/50 bp single-end reads.

Differential expression analysis of the experimental groups was carried out utilizing the DESeq R package (version 1.10.1). DESeq employs a statistical model based on the negative binomial distribution to determine differential expression in digital gene expression data. The resulting P-values underwent adjustment using the Benjamini and Hochberg method to control the false discovery rate. Genes with an adjusted P-value < 0.05, as identified by DESeq, were considered as differentially expressed. Gene Ontology (GO) enrichment and Kyoto Encyclopedia of Genes and Genomes (KEGG) pathway analysis of the identified differentially expressed genes were conducted using the Metascape platform, accessible at http://metascape.org/gp/index.html 95.

To evaluate the correlation between mTOR, Lipin1 and WAT-browning associated genes in human sWAT, we used RNA-seq data sets from the Genotype-Tissue Expression project (archived at http://www.genenetwork.org/). Significant enrichment of differentially expressed genes was determined by considering P-values less than 0.05.

### Cell culture

3T3-L1 cells were cultured to confluence in Dulbecco's modified Eagle's medium (DMEM, Thermo) containing 10% (vol/vol) fetal bovine serum (FBS, Biological Industries), with the medium changed every 2 d at 37 °C in a 5 % CO_2_ incubator. At 2 d after cell confluence, differentiation was initiated by adding differentiation medium 1 [0.5 mM 3-isobutyl-1-methylxanthine (IBMX), 0.25 μM dexamethasone, 1 μg/mL insulin in DMEM containing 10% (vol/vol) FBS].

### Primary cell isolation and culture

Fresh human subcutaneous adipose tissue samples were procured from the abdominal fat pads, usually during intra-abdominal laparoscopic surgeries of patients undergoing bariatric (BMI ≥ 28 kg/m²) or nonbariatric procedures. These tissue samples were promptly placed on ice and transported to the laboratory within 20 minutes to maintain their integrity. Subsequently, the tissues were minced and subjected to digestion in Hank's Balanced Salt Solution (HBSS) supplemented with 4% fatty-acid free bovine serum albumin (BSA), 2 mg/mL collagenase B, and 1 mg/mL soybean trypsin inhibitor. The tissue digestion process occurred over a duration of 30 minutes at a temperature of 37 °C. The resultant digested solution was then filtered through a 40 μm cell strainer and subsequently centrifuged at 500 g for 5 minutes. The resulting pellet, known as the Stromal Vascular Fraction (SVF), containing preadipocytes, was resuspended in a media composed of 90% Preadipocyte Basal Medium 2 (PBM-2), 10% fetal bovine serum (FBS), L-glutamine, gentamycin, and amphotericin. This suspension was then plated in 100 mm plates.

Cells were maintained in this media and the media was replaced every other day until the cells reached confluency. Human adipocyte differentiation was initiated by introducing a human primary adipocyte differentiation medium, which included a human differentiation cocktail composed of dexamethasone, IBMX, indomethacin, and human insulin. This was added to the culture medium according to the manufacturer's instructions. On the third day of differentiation, cells were treated with 0.25% trypsin and reseeded at a lower density (1:8 dilution) onto 12-well plates containing Type IV-collagen coated coverslips. The culture medium was changed every three days as the human primary adipocytes underwent differentiation. The differentiation medium was maintained for a period of 10 to 12 days, or until the characteristic formation of lipid droplets occurred, indicating successful adipocyte differentiation.

### RNAi-mediated gene knockdown

The nucleotide sequences for siRNAs to mouse mTOR are 5'-GAACTCGCTG ATCCAGATG-3', to mouse Lipin-1 are 5'-GGAACTCTGTAGACAGAAT-3', to human Lipin-1 are 5'-GTGGTTGACATAGAAATCA-3'. These siRNAs and control siRNA were synthesized and purified by RiboBio (China). h-mTOR-siRNA was purchased from Sigma (USA). siRNAs were transfected onto the differentiated 3T3-L1 cells or human adipocytes with Lipofectamin-2000 reagents (Invitrogen). At 24 h after infection, cells were treated with 10 nM CL316,243 or vehicle as indicated. After 24 h, oxygen consumption rates were measured or cells were harvested, and mRNA was isolated for qPCR analysis.

### Oxygen consumption rates

Primary fat Stromal Vascular Fraction (SVF) cells, derived from human subcutaneous adipose tissue, were plated into XFe 96-well cell culture microplates (Agilent #W10118) and allowed to undergo differentiation for a period of 10 days. The measurement of Oxygen Consumption Rate (OCR) was conducted at a temperature of 37 °C using an XFe analyzer manufactured by Seahorse Bioscience (USA), following the guidelines provided by the manufacturer.

During the OCR measurements, a series of compounds were administered to the cells: 5 μM oligomycin (Seahorse Bioscience, USA) to determine basal respiration, 10 μM isoproterenol, 1 μM carbonyl cyanide 4-(trifluoromethoxy) phenylhydrazone (FCCP) (Seahorse Bioscience, MA, USA) to assess uncoupled respiration, 5 μM rotenone/antimycin A (Rot/AA) (Seahorse Bioscience, USA) to measure non-mitochondrial respiration.

Relative OCR was calculated as follows: Basal OCR: Subtract OCR measured after antimycin addition from the basal OCR. Uncoupled Respiration: Subtract OCR measured after oligomycin addition from the OCR after FCCP addition. Maximal Respiration: Subtract OCR measured after antimycin addition from the OCR after FCCP addition. After completing the OCR measurement, the cells were lysed and the total double-stranded DNA (dsDNA) content per well was determined using the Quant-iT PicoGreen dsDNA Assay Kit (Thermo Fisher). All the calculated rates were normalized to 50 ng of dsDNA 96.

### MitoTracker staining

Mitochondria within the cells were labeled using the mitochondria-specific dye MitoTracker Deep Red (Life #M22426), following the instructions provided by the manufacturer. The dye was used at a final concentration of 50 nmol, and the cells were incubated with the dye for a period of 30 minutes before proceeding to visualization. To stain the cell nuclei, DAPI (Sigma #D9542-5MG) was employed. Fluorescent microscopy was conducted on live cells, using a Leica DMIRB inverted microscope, to observe the labeled mitochondria and stained nuclei.

### Bodipy staining

Cellular neutral lipid droplets of adipocytes grown in 6-well plates were stained with Bodipy (Life #D3922) following manufacturer's instruction. At least four randomly chosen areas were captured using a Leica DMIRB inverted microscope.

### Adeno-associated virus injection

Mice were anesthetized by isoflurane and placed in a prone position. All rAAV-shRNAs were purchased from Vigen Biosciences (China). shRNA sequences were as follows:

mouse mTOR: GGCAGAACTCGCTGATCCAGATGACATACATCTGTGGCTTCACTATGTCATCTGGATCAGCGAGTTT;

mouse Lipin-1: GGCAGGAACTCTGTAGACAGAATCAGTACATCTGTGGCTTCACTACTGATTCTGTCTACAGAGTTCT.

Aseptic procedures were followed to prepare the skin overlying the inguinal fat pad. An incision was made to expose the inguinal fat pad. The virus, previously diluted in sterile PBS at a concentration of 2.0×10^10 viral genomes (vg) per 20 μl for each mouse, was then injected at various sites within the inguinal fat pad. The injections were performed using a 0.3 cc insulin syringe with a 31G needle. Following the injection process, the incisions were carefully closed using surgical clips.

### Statistical analysis

Where indicated, data are expressed as mean ± standard error of means (S.E.M.). Statistical analysis was performed using SPSS (Windows version 26, IBM Analytics) or GraphPad Prism (Windows version 8.0, GraphPad Software), with a P-value of less than 0.05 considered significant. Data distribution was assessed using the Kolmogorov-Smirnov test. Statistical significance was evaluated using the unpaired two-tailed Student's t-test for comparisons between two groups. In cases where one-way ANOVA or two-way ANOVA was utilized, post hoc tests were conducted using Tukey's multiple comparisons. The p-values resulting from these tests were denoted on graphs using single asterisks (*p < 0.05) and double asterisks (**p < 0.01). For each figure, the sample sizes (n), the specific statistical tests employed, and the corresponding p-values were provided in the respective figure legend.

## Supplementary Material

Supplementary figures and tables.
