# Supplementary material for: βAR-mTOR-lipin1 pathway mediates PKA-RIIβ deficiency-induced adipose browning
Source: Theranostics. 2024 Aug 26;14(13):5316–35. doi: 10.7150/thno.97046 (PMC11388065; doi:10.7150/thno.97046)
Supplement: Supplementary file 1 — Supplementary figures and tables. [file thnov14p5316s1.pdf]

Supplementary Materials for

βAR-mTOR-lipin1 pathway mediates PKA-RIIβ deficiency-induced adipose browning

Bingwei Wang *et al*.

*Corresponding author. Email: rmzheng@pku.edu.cn

This PDF file includes:

Figures. S1 to S7

Tables S1 to S2

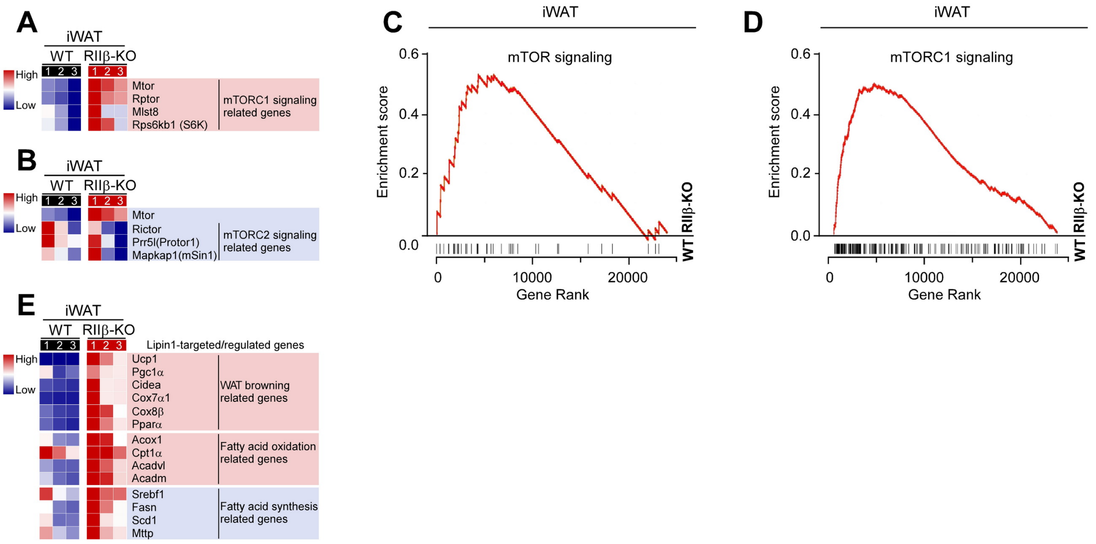

Figure S1. Increased sympathetic activity in WAT of RIIβ-KO mice.

(A) Heatmap depicting differentially expressed genes associated with mTORC1 signaling in iWAT of RIIβ-KO mice and WT mice. **(B)** Heatmap depicting differentially expressed genes associated with mTORC2 signaling in iWAT of RIIβ-KO mice and WT mice. **(C, D)** GSEA shows that the gene set related to mTOR signaling and mTORC1 signaling are significantly upregulated in iWAT of RIIβ-KO mice. **(E)** Heatmap depicting genes associated with Lipin1 signaling in iWAT of RIIβ-KO mice and WT mice. WT n = 3; RIIβ-KO n = 3. Data from Figure 2A.

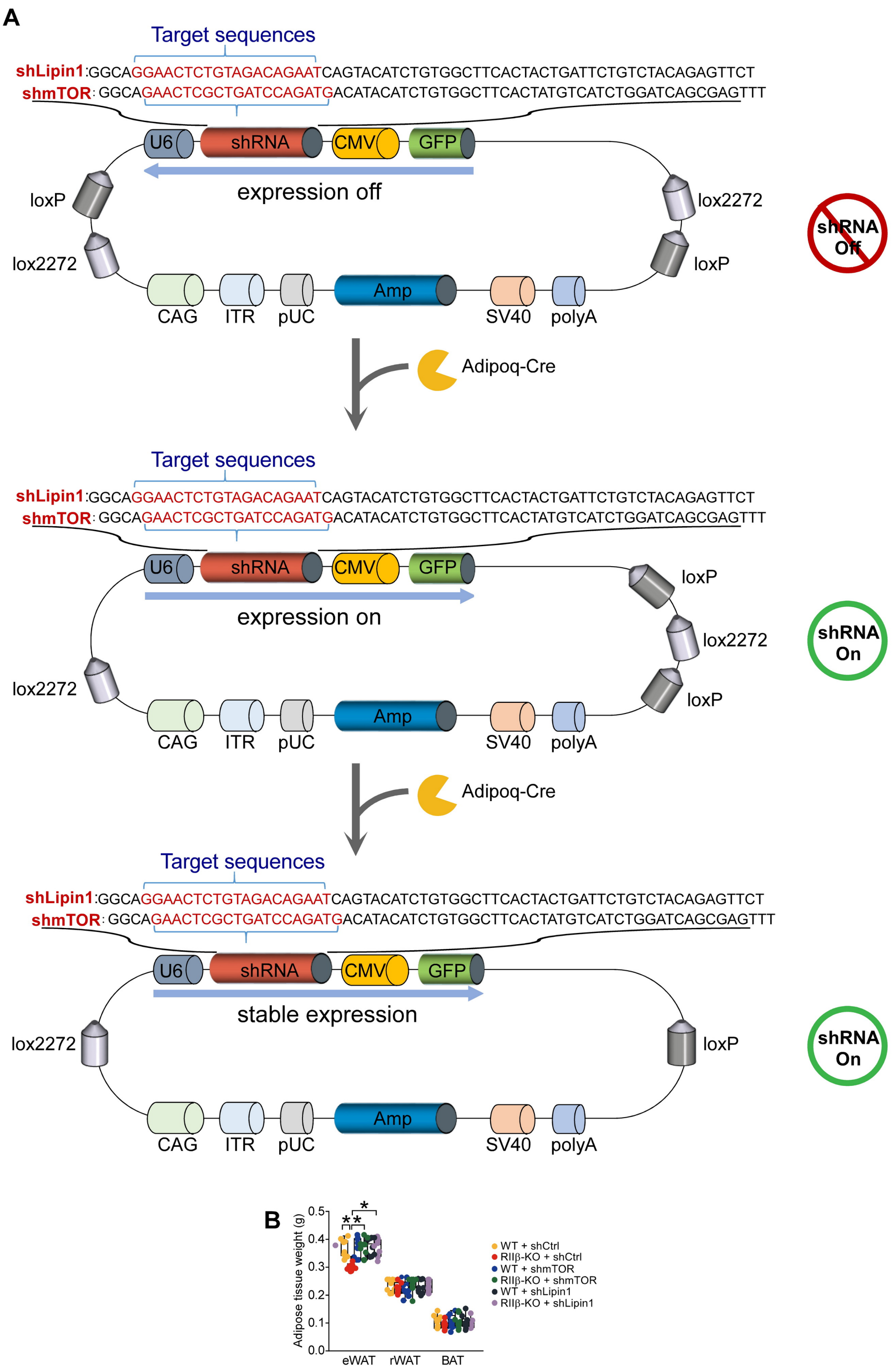

Figure S2. AAV-delivered and adipoq-Cre-mediated specific expression of shRNA in white adipocytes.

(A) Construction of the rAAV-shRNA-mTOR and rAAV-shRNA-Lipin-1 vector. (B) Fat pad weight.

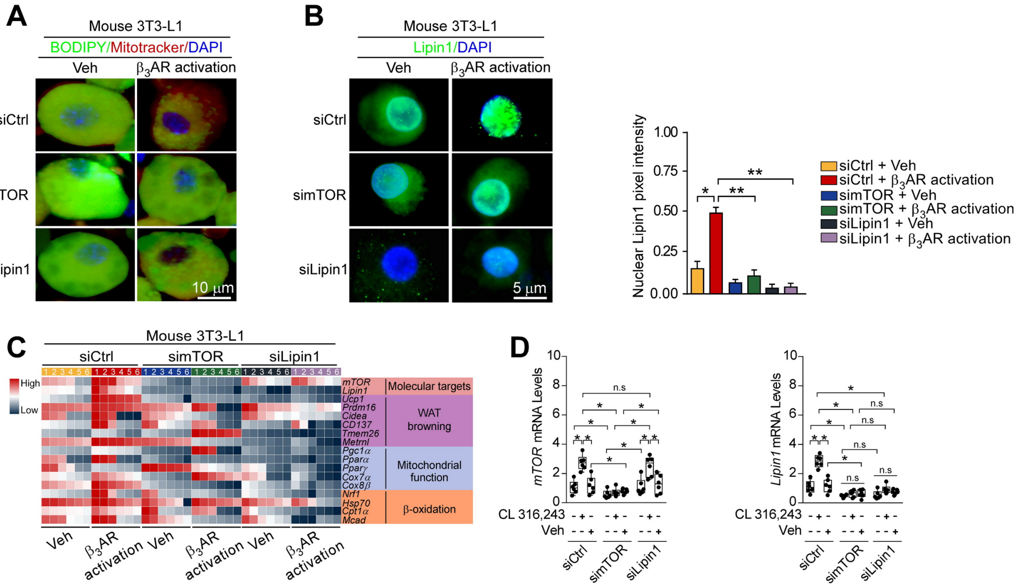

Figure S3. Knockdown of mTOR and lipin1 diminishes WAT browning-related responses in mouse 3T3-L1 adipocytes.

(A) Representative fluorescence images of BODIPY (green) and Mitotracker (red) in mouse 3T3-L1 adipocytes. Scale bar indicates 10 μm. **(B)** Representative immunofluorescence images of lipin1 in mouse 3T3-L1 adipocytes, and quantified nuclear lipin1 pixel intensity. **(C)** Heatmap shows mRNA levels of WAT browning associated genes in mouse 3T3-L1 adipocytes. siCtrl Veh n = 6; siCtrl β3AR activation n = 6; simTOR Veh n = 6; simTOR β3AR activation n = 6; siLipin1 Veh n = 6; siLipin1 β3AR activation n = 6. **(D)** RT-qPCR shows efficient knockdown of mTOR or lipin1. P values were determined by two-way ANOVA followed by Tukey's multiple comparisons test. *P < 0.05.

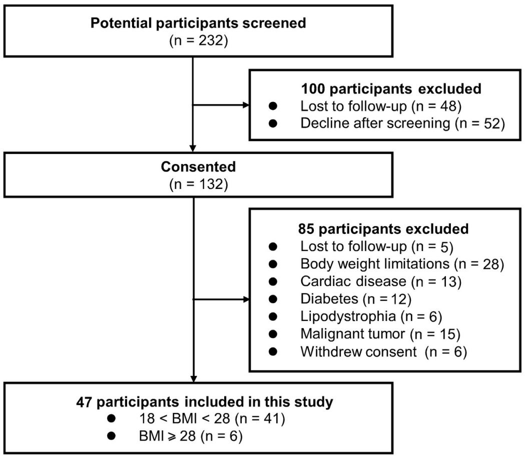

Figure S4. Flow diagram summarizing the recruitment strategy.

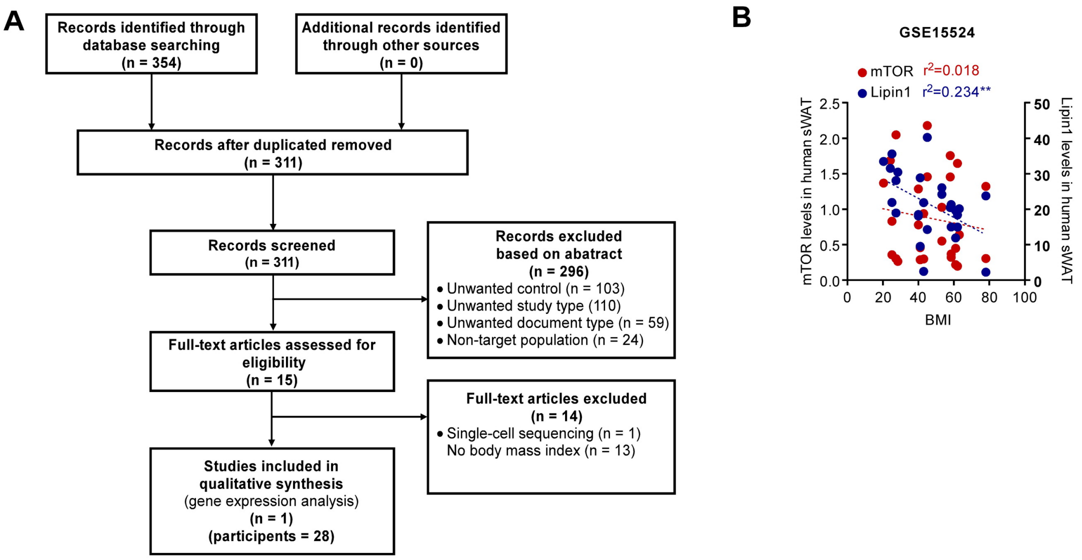

Figure S5. Flow diagram indicating the study selection process for correlation analysis.

(A) The diagram of selecting GSE datasets. (B) Correlation between mTOR and lipin1 expression levels in sWAT and BMI in humans. The genes expression levels and BMI were using geographic data sets (GSE15524).

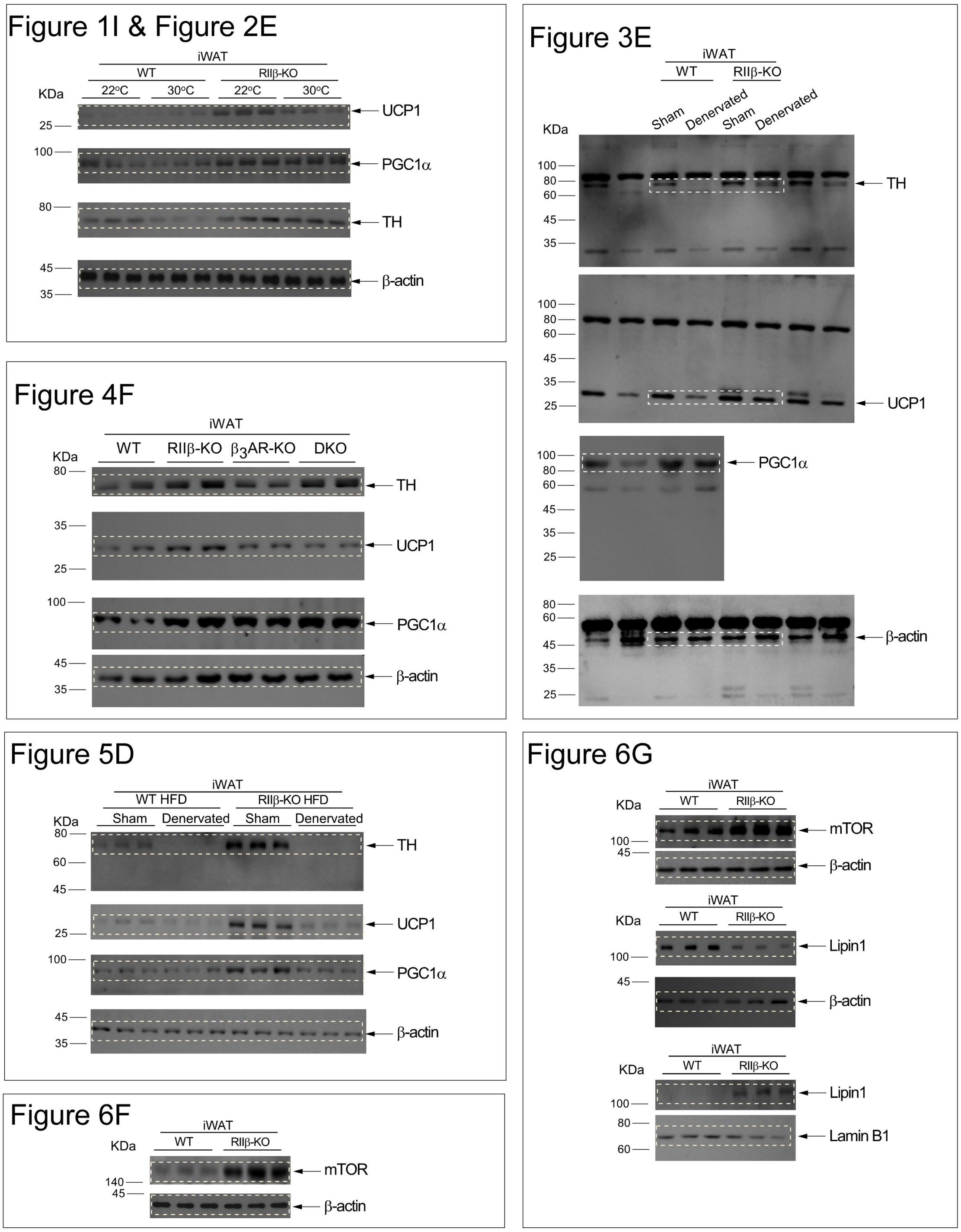

Figure S6. Full immunoblots relating to Figure 1 to Figure 6.

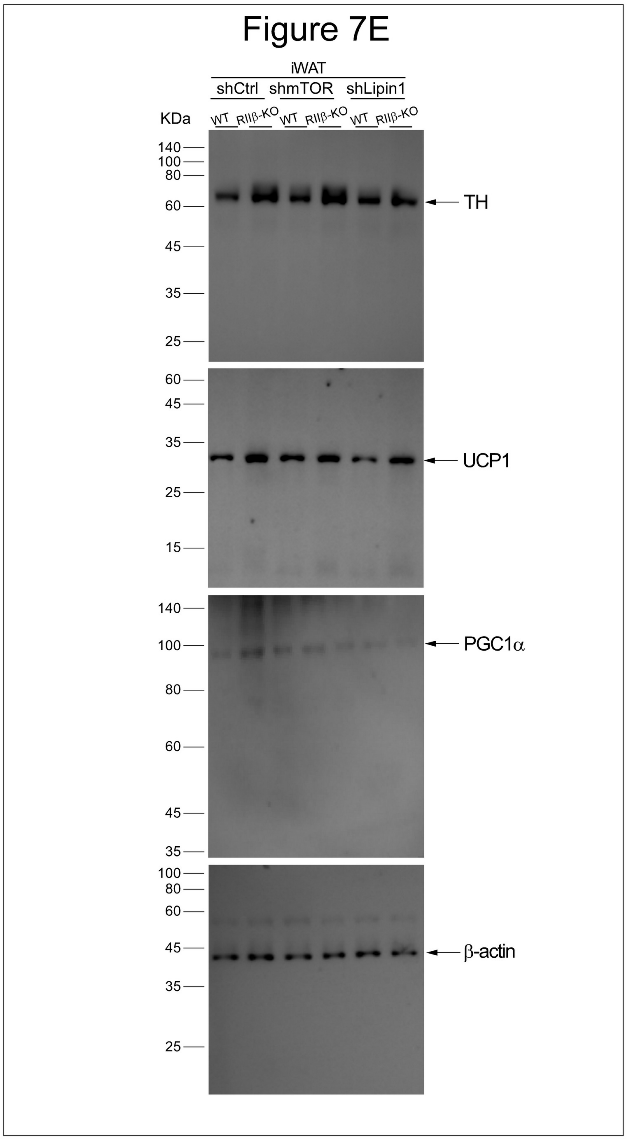

Figure S7. Full immunoblots relating to Figure 7.

Table S1: Key resources table

REAGENT	SOURCE	IDENTIFIER
Rabbit polyclonal to UCP1	Abcam	ab10983
Rabbit polyclonal to UCP1	Bioss	bs-1925R
Rabbit polyclonal to Pgc1α	Santa Cruz Biotechnology	sc-13067
Rabbit polyclonal to TH	Millipore	AB152
Rabbit polyclonal to Adrb3	Invitrogen	PA5-50914
Rabbit polyclonal to mTOR	Bioss	bs-1992R
Rabbit polyclonal to Lipin-1	Cell Signaling Technology	5195
Mouse monoclonal to β-actin	Sigma-Aldrich	A5316
Anti-rabbit IgG/Alexa Fluor 488	Bioss	bs-02950-AF488
HRP AffiniPure Goat Anti-Rat IgG(H+L)	Biodragon	BF03008
HRP AffiniPure Goat Anti-Mouse IgG(H+L)	Biodragon	BF03001
6-hydroxydopamine (6-OHDA)	Sigma-Aldrich	H4381
TranZol up	TransGen Biotech	ET111-01
TransScript one-step gDNA removel and cDNA synthesis Super MiX	TransGen Biotech	AT311-03
TransStart Top Green qPCR Super Mix	TransGen Biotech	AQ141-04

Table S2: Primers Used in This Study

Primer	Forward Primer 5'-3'	Reverse Primer 5'-3'
mTOR	AATACGCCATGAAACACTTCG	GGTCTTCCTTGTTTGTGTCCA
Lipin1	CGCCAAAGAATAACCTGGAA	TGAAGACTCGCTGTGAATGG
Ucp1	ACTGCCACACCTCCAGTCATT	CTTTGCCTCACTCAGGATTGG
Prdm16	CAGCACGGTGAAGCCATTC	GCGTGCATCCGCTTGTG
Cidea	TGCTCTTCTGTATCGCCCAGT	GCCGTGTTAAGGAATCTGCTG
CD137	CGTGCAGAACTCCTGTGATAAC	GTCCACCTATGCTGGAGAAGG
Tmem26	ACCCTGTCATCCCACAGAG	TGTTTGGTGGAGTCCTAAGGTC
Metrnl	CTGGAGCAGGGAGGCTTATTT	GGACAACAAAGTCACTGGTACAG
Pgc1α	AGCCGTGACCACTGACAACGAG	GCTGCATGGTTCTGAGTGCTAAG
Pparα	GGGTACCACTACGGAGTTCACG	CAGACAGGCACTTGTGAAAACG
Pparγ	GTGCCAGTTTCGATCCGTAGA	GGCCAGCATCGTGTAGATGA
Cox7α	CAGCGTCATGGTCAGTCTGT	AGAAAACCGTGTGGCAGAGA
Cox8β	GAACCATGAAGCCAACGACT	GCGAAGTTCACAGTGGTTCC
Nrf1	CAGCAACCCTGATGGCACCGTGTC	GGCCTCTGATGCTTGCGTCGTCTG
Mcad	ATGACGGAGCAGCCAATGAT	TCGTCACCCTTCTTCTCTGCTT
Cpt1α	TGGCATCATCACTGGTGTGTT	GTCTAGGGTCCGATTGATCTTTG
HSP70	TGGTGCTGACGAAGATGAAG	AGGTCGAAGATGAGCACGTT
Gapdh	AGGTCGGTGTGAACGGATTTG	TGTAGACCATGTAGTTGAGGTCA
18S	GCTTAATTTGACTCAACACGGGA	AGCTATCAATCTGTCAATCCTGTC
hmTOR	ATGCTTGGAACCGGACCTG	TCTTGACTCATCTCTCGGAGTT
hLipin-1	CCAGCCCAATGGAAACCTCC	AGGTGCATAGGGATAACTTCCTG
hAdrb3	GACCAACGTGTTCGTGACTTC	GCACAGGGTTTCGATGCTG
hAdrb2	TTGCTGGCACCCAATAGAAGC	CAGACGCTCGAACTTGGCA
hAdrb1	ATCGAGACCCTGTGTGTCATT	GTAGAAGGAGACTACGGACGAG
hDio2	TCCAGTGTGGTGCATGTCTC	CTGGCTCGTGAAAGGAGGTC

Primer	Forward Primer 5'-3'	Reverse Primer 5'-3'
hPgc1α	TCTGAGTCTGTATGGAGTGACAT	CCAAGTCGTTCACATCTAGTTCA
hUcp1	GGTAGTATGCAAGAGAGGTGT	CCTAATGGTACTGGAAGCCTG
hUcp2	CCCCGAAGCCTCTACAATGG	CTGAGCTTGGAATCGGACCTT
hUcp3	TGTTTTGCTGACCTCGTTACC	GACGGAGTCATAGAGGCCGAT
hPrdm16	CGAGGCCCCTGTCTACATTC	GCTCCCATCCGAAGTCTGTC
hCidea	TTATGGGATCACAGACTAAGCGA	TGCTCCTGTCATGGTTGGAGA
hTmem26	ATGGAGGGACTGGTCTTCCTT	CTTCACCTCGGTCACTCGC
hZic1	CACGCGGGACTTTCTGTTC	TGCCCGTTGACCACGTTAG
hPparα	ATGGTGGACACGGAAAGCC	CGATGGATTGCGAAATCTCTTGG
hCycs	CTTTGGGCGGAAGACAGGTC	TTATTGGCGGCTGTGTAAGAG
hCited	CCTAATGGGCGAGCACATACA	GGGGTAGGGGTGATGGTTGA
hGapdh	TGTGGGCATCAATGGATTTGG	ACACCATGTATTCCGGGTCAAT
